# Non-typhoidal *Salmonella* co-infect and complicate *Plasmodium falciparum* malaria in children under-five: A prospective cohort study on clinical presentation and outcome in Kisantu district hospital, DR Congo

**DOI:** 10.1371/journal.pntd.0014457

**Published:** 2026-06-24

**Authors:** Bieke Tack, Daniel Vita, Jules Mbuyamba, José Nketo, Emmanuel Ntangu, Marie-France Phoba, Aimée Luyindula, Gaëlle Nkoji, Hornela Vuvu, Anne-Sophie Heroes, Justin Im, Birkneh Tilahun Tadesse, Mohamadou Siribie, Hyon Jin Jeon, Florian Marks, Liselotte Hardy, Erika Vlieghe, Jaan Toelen, Jan Jacobs, Octavie Lunguya

**Affiliations:** 1 Department of Clinical Sciences, Institute of Tropical Medicine, Antwerp, Belgium; 2 Department of Microbiology, Immunology and Transplantation, KU Leuven, Leuven, Belgium; 3 Department of Pediatrics, University Hospitals Leuven, Leuven, Belgium; 4 Saint Luc Hôpital Général de Référence Kisantu, Kisantu, Democratic Republic of Congo; 5 Department of Microbiology, Institut National de Recherche Biomédicale, Kinshasa, Democratic Republic of Congo; 6 Department of Medical Biology, University Teaching Hospital of Kinshasa, Kinshasa, Democratic Republic of Congo; 7 Zone de Santé Kisantu, Kisantu, Democratic Republic of Congo; 8 Faculty of Medicine, Université Protestante au Congo, Kinshasa, Democratic Republic of Congo; 9 International Vaccine Institute, Seoul, Republic of Korea; 10 Research Investment for Global Health Technology (RIGHT) Foundation, Seoul, Republic of Korea; 11 Madagascar Institute for Vaccine Research, University of Antananarivo, Antananarivo, Madagascar; 12 Cambridge Institute of Therapeutic Immunology and Infectious Disease, University of Cambridge School of Clinical Medicine, Cambridge, United Kingdom; 13 Heidelberg Institute of Global Health, University of Heidelberg, Heidelberg, Germany; 14 Faculty of Medicine and Health Sciences, University of Antwerp, Antwerp, Belgium; 15 Department of General Internal Medicine Infectious Diseases and Tropical Medicine, University Hospital Antwerp, Antwerp, Belgium; 16 Department of Development and Regeneration, KU Leuven, Leuven, Belgium; Yale School of Medicine: Yale University School of Medicine, UNITED STATES OF AMERICA

## Abstract

**Introduction:**

Non-typhoidal *Salmonella* (NTS) bloodstream infections complicate *Plasmodium falciparum* (*Pf*) malaria infections in children under-five, but bacterial co-infections are often missed due to absence of microbiological diagnosis. We compared signs/symptoms and outcome of NTS bloodstream infection, severe *Pf* malaria and NTS-*Pf* malaria co-infections.

**Methods:**

In an area with high, stable *Pf* malaria transmission (Kongo Central, DR Congo), children (>28 days- <5 years) admitted to hospital with severe febrile illness were enrolled during 18 months (NCT04473768/NCT04850677). Data (in-hospital and 1-month post-discharge) were prospectively collected.

**Results:**

NTS bloodstream infections and severe *Pf* malaria were diagnosed in 12% (331/2682) and 52% (1389/2682) of enrolled children, respectively. NTS-*Pf* co-infections occurred in 10% (264/2682) of enrolled children, *i.e., Pf* malaria co-infected 80% (264/330) of NTS bloodstream infections and NTS co-infected 6% (78/1389) of severe *Pf* malaria. In children with recent *Pf* malaria (*i.e.,* HRP2-antigen persistence with negative microscopy), NTS occurred in 32% (173/545), making recent malaria a major risk factor for NTS (OR=5.85, p < 0.001). Compared to severe *Pf* malaria, age under-two (OR=2.19), > 3 days of fever (OR=3.28) and acute malnutrition (OR=2.20-3.48) were risk factors for NTS (p < 0.001) and NTS cases more often had hypoglycemia, grunting, hepato-/splenomegaly, jaundice or altered consciousness, but overall clinical presentation was not discriminative. In-hospital NTS case fatality was high (24% versus 3% in severe *Pf* malaria), occurred within 2 days of admission in 64% of deaths, and was preceded by general danger/sepsis signs. NTS cases had slower fever resolution, more frequent in-hospital fever recurrence, longer hospital stays, and more post-discharge deaths (n = 4) than severe *Pf* malaria cases.

**Conclusion:**

NTS and *Pf* malaria frequently co-infected children under-five. Severe *Pf* malaria and NTS bloodstream infections could not be distinguished clinically, but fatality rates were higher in NTS. Low thresholds for empirical NTS antibiotics and early danger sign recognition triggering sepsis care might improve outcome.

## Author summary

We compared non-typhoidal *Salmonella* (NTS) bloodstream infections and severe *Plasmodium falciparum* (*Pf*) malaria in children under five years in a high malaria transmission area in DR Congo.

Among 2682 children hospitalized severe febrile illness, we found that one in eight had NTS bloodstream infection. NTS-*Pf* malaria co-infection was common—80% of children with NTS bloodstream infections also had *Pf* malaria, while 6% of severe *Pf* malaria cases had concurrent NTS infection. Notably, children with recent *Pf* malaria (HRP2-antigen presence despite negative microscopy) were significantly more likely to have a NTS bloodstream infection.

Clinical features alone could not distinguish between NTS bloodstream infection or *Pf* malaria (co-) infections. However, children with NTS infection were more often <2 years, more often malnourished, and presented more often with prolonged fever and signs of severe systemic illness, such as grunting, hypoglycemia or altered consciousness. Compared to severe *Pf* malaria, NTS cases had a substantially higher in-hospital case fatality rate (24% vs. 3%), with most deaths occurring within 48 hours of admission.

Our findings highlight the difficulty of diagnosing NTS bloodstream infections in malaria-endemic settings. They underscore the need for early empirical antibiotic treatment in children with severe febrile illness, irrespective of malaria status.

## Introduction

In children under-five in sub-Saharan Africa, non-typhoidal *Salmonella* (NTS) frequently cause severe febrile illness, including a third of community-acquired bloodstream infections [1–4]. Each year, approximately 420,000 NTS infections occur per year in sub-Saharan Africa, accounting for 66,500 deaths (all-age case fatality 15.8%), from which >40% of deaths are in children under-five [5]. *Plasmodium falciparum* (*Pf*) malaria is diagnosed more frequently with an estimated yearly count of 233 million African cases, but has a lower case fatality ratio (0.2%) resulting in approximately 580,000 deaths per year, from which approximately 60% are in children under-five [6].

The circulation of virulent human-adapted NTS strains, local host susceptibility, and difficult diagnostic and therapeutic management determine the high incidence and mortality of invasive NTS infections in sub-Saharan Africa [7]. Serotypes causing invasive NTS infection in sub-Saharan Africa are the same as those globally causing self-limiting diarrhea, *i.e., Salmonella* Typhimurium and Enteritidis [8]. However, genotypic NTS lineages circulating in sub-Saharan Africa are more virulent and adapted to the human host, which probably creates a human reservoir of infection [8,9]. Furthermore, severe or recurrent *Pf* malaria infections, hemolytic anemia, malnutrition, and HIV infection impair the immune response of children under-five and predispose to NTS bloodstream infections [8]. Some of the high malaria burden countries, including the Democratic Republic of Congo (DR Congo), have increasing numbers of malaria cases and numbers, which has been associated with increasing numbers of invasive NTS cases [10].

Lastly, NTS bloodstream infections are often overlooked. Blood cultures are required for diagnosis, but they are rarely available in sub-Saharan Africa [11]. In addition, diagnostic confusion occurs due to NTS – *Pf* malaria co-infections, clinical similarities with severe *Pf* malaria, and clinical similarities with other severe bacterial infections (*e.g.,* pneumonia) [8]. Therefore, the World Health Organization (WHO) recommends administering broad-spectrum antibiotics in all children admitted with severe *Pf* malaria in areas endemic for invasive NTS infections [12]. However, NTS are frequently resistant to commonly used broad spectrum antibiotic regimens for severe bacterial infections, *e.g.,* ampicillin + gentamicin or ceftriaxone [13]. Better clinical recognition is hence required for early appropriate antibiotic treatment and reduction of NTS-related case fatality.

In this large, prospective, hospital-based cohort study in DR Congo, we aimed to compare the clinical presentation, in-hospital clinical evolution, and post-discharge outcome of children under-five with severe *Pf* malaria, NTS bloodstream infection, and NTS – *Pf* malaria co-infections. We also compared with non-NTS bloodstream infection and other severe febrile illness etiology. Secondly, we identified danger signs associated with in-hospital death in children with NTS bloodstream infections.

## Methods

### Ethical statement

Caretakers of eligible children gave written informed consent before enrollment. If the caretaker was not the parent/legal guardian of the child, we accepted informed consent of the caretaker who actually accompanied the child. In this case, we asked the caretaker to inform the parents/legal guardian(s) that they can withdraw from the study in case of objection. The Ecole de Santé Publique de Kinshasa (144/2020, 134/2021), the Institutional Review Board of ITM (1419/20, 1483/21) and the Ethics Committee of Antwerp University (20/37/465, 21/18/236) granted ethical approval.

### Study design, period, and setting

Children were enrolled during an 18-month study period: from February 2021-January 2022, children were enrolled in a prospective, observational study focused on clinical presentation and evolution (DeNTS study; clinicaltrials.gov: NCT04473768), and from August 2021-July 2022, children were (also) enrolled in a prospective, observational follow-up study (TreNTS study, focused on antibiotic treatment; clinicaltrials.gov: NCT04850677).

Children aged >28 days - < 5 years were eligible if admitted to St. Luc Kisantu General Referral Hospital (Kisantu hospital) with severe febrile illness (fulfilling criteria for blood culture sampling, Table A in S1 Appendix). Eligibility screening, enrollment and data collection were done as soon as possible after hospital arrival, 7/7 days. Children presenting after working hours (weekdays: after 16h, weekend: after 12h) were enrolled the next morning.

Kisantu hospital functions as referral hospital for Kisantu health district (DR Congo, Kongo-Central province, ±120 km Southwest of Kinshasa) with a population-estimate of 214,780 inhabitants in 2021. Kisantu hospital applies a flat fee of 15 USD per admission, covering basic diagnostic and therapeutic management [14]. Details on the local health system and health itinerary of hospital-admitted children were published elsewhere [15]. Children enrolled in this study from February – July 2021 were also enrolled in the health itinerary study (NCT04289688), in which NTS – *Pf* malaria infections and case fatality were also discussed [15]. Kisantu district is a semi-rural area with high, stable *Pf* malaria transmission which increases in the rainy season (October-May) [10,16]. Associated with frequent *Pf* malaria, anemia, and malnutrition, many NTS bloodstream infections in children under-five occur [10,16]. HIV prevalence is relatively low (0.2% at antenatal visits) [16].

### Data collection

Table 1 summarizes data collection. Research physicians performed the physical examination and in-hospital follow-up; other data were collected by research nurses. Data were collected separately from standard patient management, except for malaria microscopy and blood culture sampling. Table B in S1 Appendix describes actions to control and assure data quality. Rainfall estimates were downloaded from Google Earth Engine (CHIRPS daily v2.0, as previously described) [10,17].

**Table 1:** Overview of data collection per time point.

When?	Enrollment = Day 0	Day 1 - Day 3	Day 3 - Discharge	Post-discharge
**Study population**	**All enrolled children**	**In-hospital follow-up of all children for 3 calendar days**	**Follow-up of children with growth in blood** culture (stop if contaminant). Follow-up of 1 age-matched control* with severe *Pf* malaria for each child with confirmed BSI followed after day 3 After January 2022**: no follow-up of control cases or non-NTS BSI	
**Data collection**	**Vital signs & anthropometry,** described in [19,30]: - Tympanic T°, HR, SpO2, weight, height, MUAC: measured with diagnostic devices - RR: counted manually	**Clinical follow-up:** - Survival - Subjective fever & T°: > Before August 2021: morning > From August 2021: morning & evening - Focal infections	**Phone call** (12–16 days after discharge): - Survival	
	**Medical history (incl prehospital care) & physical examination**				**Post-discharge** - Fever recurrence	
	**Rapid tests with capillary blood:** - Hb & Glc, as described in [19]. - Malaria antigen tests (SD BIOLINE Malaria Ag P.f./Pan test 05FK60, SD, Suwon, Korea)		Home visit *** (21–35 days after discharge): - Survival - Fever recurrence - Treatment - Readmission			
	**As part of standard patient care (venous blood):** - Malaria microscopy - Blood culture sampling	**Microbiological follow-up:** - From August 2021: control blood culture after 5 days presumed appropriate **** antibiotic NTS treatment - Microbiology from standard care			**Post-discharge**- GPS coordinates	

* Controls were selected by the research physicians among severe Pf malaria cases from the same age category (<6 months, 6- < 12 months, 12- < 24 months, ≥ 24 months) that were admitted concurrently with or soon after the NTS case.

** Timings are explained by the fact that DeNTS and TreNTS study collected slightly different data on the same study cohort. The DeNTS study lasted from February 2021-January 2022, the TreNTS study from August 2021-July 2022. Data on defervescence and post-discharge outcome were only collected from NTS cases after DeNTS study completion. Fever was only measured once daily before the start of the TreNTS study. Data from control blood cultures were only collected once the TreNTS study started.

*** During spontaneous or planned follow-up visits to Kisantu hospital, the same data (except GPS coordinates) were collected.

**** Antibiotics presumed to be appropriate according to the antibiotic susceptibility testing results obtained on-site. Control cultures were sampled as part of the TreNTS study to assess microbiological outcome.

Abbreviations: T°: temperature, HR: heart rate, SpO2: oxygen saturation, RR: respiratory rate, MUAC: mid-upper arm circumference, Hb: hemoglobin, Glc: glycemia, NTS: non-typhoidal Salmonella, BSI: bloodstream infection.

### Clinical definitions

Tachypnea, tachycardia, malnutrition, anemia, and hypoglycemia were defined as in the WHO pocketbook for hospital care of children [18,19]. Hemoglobin levels of children with in-hospital blood transfusion before enrollment were corrected with -2 g/dl to estimate pre-transfusion hemoglobin [20,21]. Prostration is inability to sit if age ≥ 1 year and inability to breastfeed if age <1 year [22]. Current *Pf* malaria was defined as positive *Pf* malaria microscopy. Recent *Pf* malaria was defined as negative *Pf* malaria microscopy but positive *Pf*-HRP2 antigen on rapid test [23,24]. Mixed infections of non-*falciparum* malaria with *Pf* malaria was classified as *Pf* malaria. Severe *Pf* malaria was defined according to WHO criteria [12] with small adaptations for criteria that were locally unfeasible to assess (Table C in S1 Appendix). NTS – *Pf* malaria co-infections were defined as NTS bloodstream infection with current/recent *Pf* malaria. Other febrile illness refers to severe febrile illness of other, non-specified causes (no bloodstream infection, no severe *Pf* malaria). Fever resolution was defined as absent subjective fever (reported by caretaker) and axillary/tympanic temperature ≤37.5°C for 2 days. Fever recurrence was defined as recurrence of subjective fever or axillary/tympanic temperature >38.0°C after fever resolution (until hospital discharge for in-hospital fever recurrence, between discharge and the end of post-discharge follow-up for post-discharge fever recurrence).

### Laboratory work-up

Free-of-charge blood cultures were routinely sampled on admission and worked-up on-site as part of a national blood culture surveillance network, organized by the Institut Nationale de Recherche Biomédicale (INRB, Kinshasa, DR Congo) and Institute of Tropical Medicine (ITM, Antwerp, Belgium), according to previously published procedures (manual incubation, biochemical identification, disk diffusion for antibiotic susceptibility testing) [25–28]. Reference testing (MALDI-TOF MS identification (Bruker, Billerica, USA), *Salmonella* serotyping, broth microdilution for antibiotic susceptibility testing) was performed at ITM (Table B in S1 Appendix). Results from antibiotic susceptibility testing and treatment are reported in more detail in a separate manuscript submitted elsewhere for publication.

Until March 4, 2021 and from August 9, 2021 onwards, blood cultures were also sampled in selected health centers in Kisantu district, respectively as part of the SETA [29] and TyVECO (NCT05562102) surveillance study. If children with a blood culture sampled in participating health centers arrived in Kisantu hospital, no new blood culture was sampled. The SETA/TyVECO surveillance is part of a typhoid conjugate vaccine study (Typbar TCV, Bharat Biotech, Hyderabad, India; NCT05119426) with mass vaccination of children in Kisantu district from February 10, 2022 onwards. Blood cultures from SETA/TyVECO were worked up in Kisantu hospital and reference tested according to the same procedures [25,26].

### Antimicrobial treatment

Intravenous third generation cephalosporins were used as standard-of-care empirical antibiotics, with switch to ciprofloxacin when Gram-negative rods were identified from the blood culture. Oral azithromycin was used to treat third generation cephalosporin resistant and fluoroquinolone non-susceptible NTS bloodstream infections. Malaria was treated with artesunate IV followed by oral artemisinin combination therapy, according to WHO recommendations [12].

### Data-analysis

Data were analyzed in R 4.2.2 (R Foundation for Statistical Computing, Vienna, Austria). Proportions were compared with odds ratios (OR) and their 95% confidence interval (95% CI), and chi-square/fisher exact test. Continuous variables were compared based on the median, 25^th^ & 75^th^ percentiles (P25-P75), and Wilcoxon rank-sum test. Survival analysis (Kaplan-Meier curves, Cox regression for hazard ratios (HR)) was performed with R-packages ‘survival’ and ‘survminer.’ Figures were made with ‘ggplot2’ and adapted in PowerPoint (Microsoft Corporation, Redmond, USA). The map was created with R-packages ‘sf‘ & ‘ggsn’, with base layer data available open access at http://www.rgc.cd/ [14].

## Results

### Enrollment and study population

**Fig 1:**
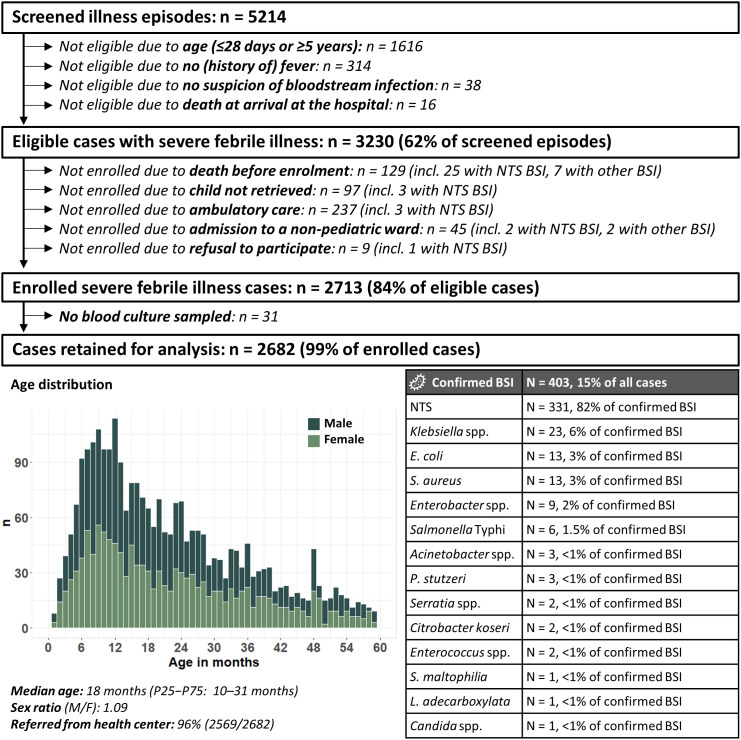
Overview of study enrollment and age, sex, and blood culture results. Abbreviations: BSI: bloodstream infection, NTS: non-typhoidal *Salmonella,* M/F: male/female, *E. coli: Escherichia coli*, *S. aureus: Staphylococcus aureus, P. stutzeri: Pseudomonas stutzeri, S. maltophilia: Stenotrophomonas maltophilia, L. adecarboxylata: Leclercia adecarboxylata.*

A total of 2682 children with severe febrile illness were enrolled and retained for analysis, they represented 83% of eligible cases (Fig 1). Among them, 43% (n = 1145) were immediately enrolled at hospital arrival and 99.8% (n = 2676) were enrolled within 24 hours. Over the 18-month study period, 12% (310/2682) were enrolled more than once (2 episodes: n = 255; 3 episodes: n = 43; 4 episodes: n = 10; 5 episodes: n = 2), with a median 90-day interval between successive episodes (P25-P75: 47–169 days). Fig 1 summarizes enrollment.

Almost all (96%) cases were referred from a health center and the median age was 18 months (Fig 1). Most (99.6%, 2670/2682) caretakers knew the vaccination status of the child and 89% (2383/2670) reported the child as fully vaccinated for their age. Sickle cell disease was reported as medical history in 0.5% (13/2682); HIV was never reported. Almost half (45%, 1211/2662) of cases had been admitted at least once in a hospital or health center during the preceding 12 months. Almost half (45%, 1212/2676) of cases had already been transfused blood at least once in their life.

### Non-typhoidal *Salmonella* bloodstream infections and *Pf* malaria co-infections

Blood cultures were sampled on the day of admission in 96% (2587/2682) and pathogens were detected in 15% of them (403/2682; Fig 1). This high blood culture positivity translated in a number needed to treat of seven severe febrile illness cases to cover one culture-confirmed bloodstream infection. Seven bloodstream infections were mixed (5 NTS + *Klebsiella pneumoniae*, 2 NTS + *Acinetobacter* spp.). Almost all isolates (96%, 395/410) were reference tested.

Non-typhoidal *Salmonella* were isolated in 12.3% (331/2682) of severely febrile patients, representing 82% (331/403) of culture-confirmed bloodstream infections and had a median time-to-positivity of 1 day (P25-P75: 1–2 days). Reference serotype and antibiotic susceptibility data were available for 98% (325/331) of NTS. Most (83%, 271/325) were serotype Typhimurium, from which 40% (108/271) were O5-antigen negative (a.k.a. Typhimurium variant Copenhagen) [25]. All other NTS were serotype Enteritidis (17%, 54/325). Multidrug resistance, *i.e.,* co-resistance to ampicillin, cotrimoxazole and chloramphenicol, occurred in 85% (275/325) of NTS bloodstream infections. Third generation cephalosporin resistance and fluoroquinolone non-susceptibility were respectively detected in 74% (242/325) and 72% (233/325) of NTS isolates. Azithromycin resistance was rare (2%) and there was no carbapenem resistance. Table D in S1 Appendix reports cumulative antibiograms of all pathogens isolated from blood cultures at admission.

**Fig 2:**
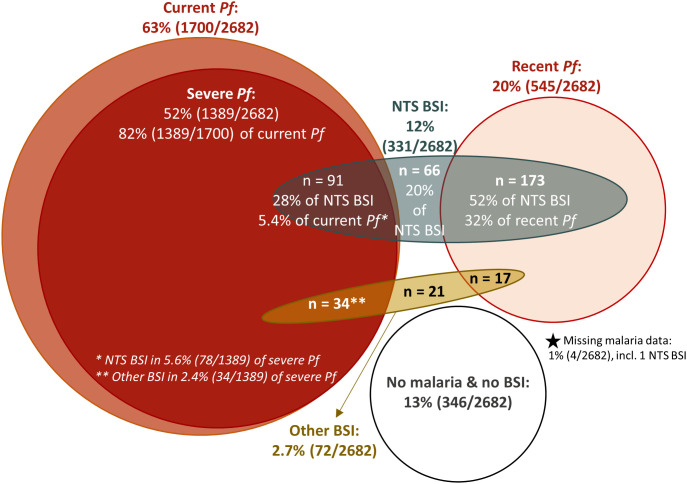
Graphical representation of *Plasmodium falciparum (Pf)* malaria co-infections in non-typhoidal *Salmonella* (NTS) and other bloodstream infections (BSI). Surface areas are proportional to counts. Unless otherwise specified, percentages refer to the proportion of all cases.

Current *Pf* malaria was diagnosed in 63% (1700/2682) of enrolled children, from which 82% (1389/1700) had severe *Pf* malaria (Fig 2). Among severe *Pf* malaria cases, co-infection with NTS was observed in 5.6% (78/1389) and co-infection with other blood culture pathogens was observed in 2.4% (34/1389). One in three (32%, 173/545) children with recent *Pf* malaria was co-infected with NTS, while recent *Pf* malaria co-infection with other blood culture pathogens was rare (3%, 17/545). Recent *Pf* malaria was therefore a strong risk factor for NTS bloodstream infection (OR 5.85 [4.59-7.46] for NTS versus no NTS bloodstream infection). Overall, 80% (264/330) of NTS bloodstream infections were *Pf* malaria co-infected. In those co-infected with current *Pf* malaria, 46% (42/91) had parasite densities <5000/µL, 19% (17/91) had parasite densities >50.000/µL, and median parasite density was significantly lower than in severe *Pf* malaria (p < 0.001, Fig A in S1 Appendix). Overall, non-*falciparum* malaria was rare (9 *P. malariae* and 6 *P. ovale*). Two mixed infections with *Pf* malaria (1 with *P. ovale*, 1 with *P. malariae*) were classified as *Pf* malaria.

### Seasonal and spatiotemporal NTS dynamics

Severe *Pf* malaria and NTS with/without *Pf* malaria co-infection increased during the rainy season (Fig C in S1 Appendix). Severe *Pf* malaria without bloodstream infection cases and case fatality peaked in the early rainy season (October – December). The NTS cases and deaths (irrespective of *Pf* malaria co-infection) peaked later (December – March), and the absolute number of NTS deaths and the NTS case fatality ratios were strikingly high in February 2021 and February 2022. There were no apparent differences in seasonal dynamics between serotypes (Fig D in S1 Appendix).

**Fig 3:**
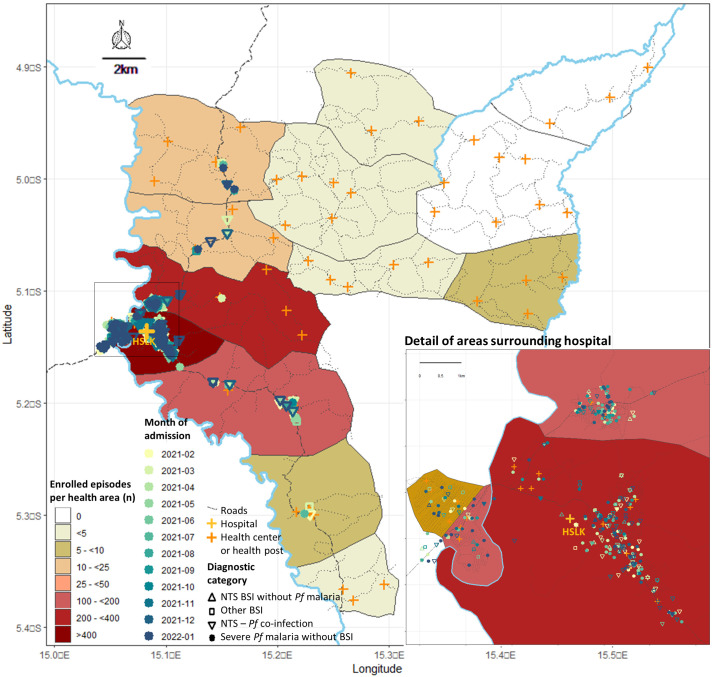
Spatiotemporal distribution of non-typhoidal *Salmonella* (NTS) and other-than-NTS bloodstream infections (BSI) vs. age-matched controls with severe *Plasmodium falciparum (Pf)* malaria, based on GPS coordinates of post-discharge home visits. The map was created with R-packages ‘sf‘& ‘ggsn’ with open access base layer data at https://data.humdata.org/dataset/zones-de-sante-rdc [14].

Assessment of spatiotemporal clustering of NTS based on GPS coordinates from home visits revealed that some children admitted with NTS bloodstream infection in the same month, lived close to each other (Fig 3). However, the overall geographical NTS distribution reflected the geographical variation in enrollment frequency and clustering occurred mostly along the main roads.

### Risk factors & clinical presentation of NTS bloodstream infection

Table 2 compares the risk factors and clinical presentation of the following categories: NTS bloodstream infection (with and without *Pf* malaria co-infection), severe *Pf* malaria without bloodstream infection, other bloodstream infections and other febrile illnesses. Additional analyses comparing severe *Pf* malaria with NTS–*Pf* malaria co-infection and NTS without *Pf* malaria co-infection as separate groups are reported in Table E in S1 Appendix and provided similar results. Compared to children with severe *Pf* malaria without bloodstream infection, NTS cases were younger. The median age of NTS cases was 15 (P25-P75: 10–23) months. Three-quarters (76%; 252/331) were <2 years, but few (8%, 27/331) were <6 months old. NTS cases were twice more often malnourished than children with severe *Pf* malaria (33% vs. 16%) and, from all children with acute malnutrition, one in five (110/532) had an NTS bloodstream infection. Compared to severe *Pf* malaria without bloodstream infection, the proportion of NTS cases who received prehospital antibiotics (44% versus 29%), antimalarials (48% versus 32%), and transfusions (14% versus 4%) was higher. The proportion of NTS cases presenting to hospital with >3 days of fever was higher (58%) compared to severe *Pf* malaria cases (29%).

**Table 2:** Clinical presentation according to diagnostic category comparing cases with and without non-typhoidal *Salmonella* (NTS) bloodstream infection (BSI).

	Severe *Pf* malaria without BSI (n = 1277)	NTS - *Pf* malaria co-infection (n = 264)	NTS BSI without *Pf* malaria (n = 66)	NTS BSI with/without *Pf* coinfection vs. severe *Pf* malaria without BSI		Other BSI (n = 72)	Other febrile illness (n = 999) *(no severe Pf malaria + no BSI)*
				OR [95% CI]	p-value		
**Female sex**	48.6% (n = 621)	51.1% (n = 135)	34.8% (n = 23)	0.97 [0.76–1.24]	0.856	50.0% (n = 36)	46.3% (n = 463)
**Age under 2 years**	59.2% (n = 756)	71.5% (n = 189)	93.9% (n = 62)	**2.19 [1.66–2.89]**	**<0.001**	62.5% (n = 45)	61.1% (n = 611)
**Residence in rural village**	8.8% (n = 113)	14.0% (n = 37)	16.6% (n = 11)	**1.75 [1.22–2.52]**	**0.003**	13.8% (n = 10)	10.9% (n = 109)
**Previous hospital admission** *(in 12 months before fever onset)*	46.7% (n = 596)	55.5% (n = 146)	51.5% (n = 34)	**1.37 [1.08–1.75]**	**0.012**	41.6% (n = 30)	41.9% (n = 419)
**>3 days of fever**	29.2% (n = 374)	57.1% (n = 151)	59.0% (n = 39)	**3.28 [2.55–4.20]**	**<0.001**	54.1% (n = 39)	37.1% (n = 371)
**Cough**	25.6% (n = 327)	33.3% (n = 88)	45.4% (n = 30)	**1.62 [1.25–2.09]**	**<0.001**	31.9% (n = 23)	33.7% (n = 337)
**Vomiting**	33.1% (n = 423)	30.6% (n = 81)	30.3% (n = 20)	0.89 [0.69–1.16]	0.421	41.6% (n = 30)	31.2% (n = 312)
**Diarrhea**	16.0% (n = 205)	31.4% (n = 83)	33.3% (n = 22)	**2.44 [1.85–3.21]**	**<0.001**	26.3% (n = 19)	20.7% (n = 207)
**Incapacity to eat/drink**	7.1% (n = 91)	10.9% (n = 29)	9.1% (n = 6)	**1.55 [1.03–2.33]**	**0.048**	20.8% (n = 15)	6.0% (n = 60)
**Prehospital antibiotics**	28.7% (n = 367)	40.5% (n = 107)	57.5% (n = 38)	**1.94 [1.52–2.49]**	**<0.001**	37.5% (n = 27)	41.6% (n = 416)
**Prehospital antimalarials**	32.2% (n = 412)	45.8% (n = 121)	56.0% (n = 37)	**1.93 [1.51–2.47]**	**<0.001**	31.9% (n = 23)	40.7% (n = 407)
**Prehospital transfusion**	4.4% (n = 56)	14.3% (n = 38)	12.1% (n = 8)	**3.53 [2.34–5.33]**	**<0.001**	22.2% (n = 16)	5.7% (n = 57)
**Prehospital iron supplements**	22.4% (n = 287)	28.4% (n = 75)	22.7% (n = 15)	1.29 [0.98–1.70]	0.078	29.1% (n = 21)	22.5% (n = 225)
**Moderate acute malnutrition**	10.5% (n = 135)	18.5% (n = 49)	18.1% (n = 12)	**2.20 [1.57–3.07]**	**<0.001**	13.8% (n = 10)	11.1% (n = 111)
**Severe acute malnutrition**	5.2% (n = 67)	13.6% (n = 36)	18.1% (n = 12)	**3.48 [2.34–5.19]**	**<0.001**	12.5% (n = 9)	8.8% (n = 88)
**Fever at enrollment**	42.0% (n = 537)	40.5% (n = 107)	53.0% (n = 35)	1.04 [0.82–1.33]	0.796	43.0% (n = 31)	25.5% (n = 255)
**Tachycardia at enrollment**	80.7% (n = 1031)	76.1% (n = 201)	66.6% (n = 44)	**0.69 [0.52–0.91]**	**0.012**	69.4% (n = 50)	54.3% (n = 543)
**Tachypnea at enrollment**	75.7% (n = 967)	69.3% (n = 183)	56.0% (n = 37)	**0.64 [0.49–0.83]**	**0.001**	62.5% (n = 45)	29.6% (n = 296)
**Hypoxia at enrollment**	6.2% (n = 79)	7.2% (n = 19)	18.1% (n = 12)	1.57 [1.02–2.43]	0.053	19.4% (n = 14)	6.9% (n = 69)
**Prostration**	69.4% (n = 887)	65.5% (n = 173)	59.0% (n = 39)	0.79 [0.61–1.02]	0.08	77.7% (n = 56)	32.6% (n = 326)
**Clinical dehydration**	3.0% (n = 38)	7.2% (n = 19)	9.1% (n = 6)	**2.67 [1.59–4.50]**	**<0.001**	8.3% (n = 6)	3.2% (n = 32)
**Jaundice**	4.9% (n = 63)	12.8% (n = 34)	12.1% (n = 8)	**2.81 [1.86–4.24]**	**<0.001**	9.7% (n = 7)	4.8% (n = 48)
**Not alert** *(AVPU scale: V, P or U)*	10.0% (n = 128)	14.7% (n = 39)	12.1% (n = 8)	**1.49 [1.04–2.13]**	**0.036**	25.0% (n = 18)	5.1% (n = 51)
***Convulsions*** *(before/at enrollment)*	18.0% (n = 230)	12.1% (n = 32)	15.1% (n = 10)	**0.66 [0.47–0.94]**	**0.027**	25.0% (n = 18)	9.2% (n = 92)
**Signs of meningitis** *(bombing fontanel, neck stiffness or irritability)*	3.1% (n = 39)	2.7% (n = 7)	7.6% (n = 5)	1.20 [0.62–2.31]	0.598	4.2% (n = 3)	1.5% (n = 15)
**Grunting**	18.0% (n = 231)	22.7% (n = 60)	27.2% (n = 18)	**1.40 [1.05–1.88]**	**0.028**	30.5% (n = 22)	8.0% (n = 80)
**Labored breathing** *(nasal flaring, chest/intercostal retractions)*	25.8% (n = 330)	29.9% (n = 79)	36.3% (n = 24)	1.30 [1.00–1.70]	0.059	41.6% (n = 30)	13.3% (n = 133)
**Hepatomegaly**	24.3% (n = 311)	40.1% (n = 106)	39.3% (n = 26)	**2.07 [1.61–2.67]**	**<0.001**	45.8% (n = 33)	20.3% (n = 203)
**Splenomegaly**	25.2% (n = 323)	38.6% (n = 102)	36.3% (n = 24)	**1.82 [1.41–2.36]**	**<0.001**	26.3% (n = 19)	21.7% (n = 217)
**Moderate anemia** *(Hb <=9.3 g/dl)*	75.0% (n = 958)	68.5% (n = 181)	66.6% (n = 44)	**0.56 [0.42–0.76]**	**<0.001**	51.3% (n = 37)	64.8% (n = 648)
**Severe anemia** *(Hb < 5 g/dl)*	10.1% (n = 130)	9.5% (n = 25)	1.5% (n = 1)	**0.48 [0.29–0.79]**	**0.005**	6.94% (n = 5)	2.1% (n = 21)
**Hypoglycemia** *(<45 mg/dl)*	2.5% (n = 32)	9.1% (n = 24)	9.1% (n = 6)	**3.89 [2.33–6.50]**	**<0.001**	11.1% (n = 8)	1.2% (n = 12)

Diagnostic categories were defined by confirmed bloodstream infection and *Plasmodium falciparum* (*Pf*) malaria status. Cases with unknown malaria status (n = 4) were excluded from the analysis in this table. *Reference: no acute malnutrition/no anemia.

Clinical presentation of NTS cases was not specific and multiple organ systems were affected. As in severe *Pf* malaria, two out of three NTS cases had prostration. Diarrhea was reported in a third of NTS cases versus in a sixth of severe *Pf* malaria cases but was rarely bloody (7/330 (2%) of NTS). Vomiting was not associated with NTS, but incapacity to eat/drink and clinical dehydration (*i.e.,* sunken eyes/decreased skin turgor) were, although both were present in <11% of NTS cases. Many NTS cases had respiratory signs suggestive for pneumonia or metabolic acidosis due to sepsis, *i.e.,* cough, rapid breathing, grunting or labored breathing. While severe *Pf* malaria cases more frequently (had) convulsed than NTS cases (18% vs. 13%), NTS cases more often had an altered consciousness than severe *Pf* malaria cases (14% vs. 10%). Twelve (4%) NTS cases had signs of meningitis. Hypoglycemia was strongly associated with NTS and present in 9% of NTS cases. Strong associations of jaundice, hepato- and splenomegaly with NTS cases suggested an association with (chronic) hemolytic anemia. Nevertheless, (moderate/severe) anemia was more frequent in severe *Pf* malaria than in NTS cases.

Median hemoglobin levels were also lower in severe *Pf* malaria cases than in children with NTS – *Pf* malaria co-infection and NTS without *Pf* malaria (median 7.1 g/dl, 7.4 g/dl and 8.4 g/dl, respectively; p ≤ 0.01; Fig A in S1 Appendix). However, severe anemia was much more frequent in NTS cases than in children with other bloodstream infections or other febrile illnesses (OR=4.57, 95% CI [2.52-8.29]).

Comparison of NTS bloodstream infections according to malaria status (Table F in S1 Appendix) revealed that NTS cases without *Pf* malaria were younger than children with NTS – *Pf* malaria co-infection (median age: 10 (P25-P75: 7–16) vs. 17 (P25-P75: 11–26) months, p < 0.001) and were more often hypoxic. They were not significantly more often malnourished. Children with NTS and current *Pf* malaria less often presented with fever >3 days, diarrhea, prehospital antibiotics, antimalarials or blood transfusions than NTS cases without *Pf* malaria or NTS cases with recent *Pf* malaria (Table F in S1 Appendix, OR 0.13-0.50, p ≤ 0.02). Tachycardia, tachypnea, and anemia were more frequent in NTS cases with current *Pf* malaria (Table F in S1 Appendix, 2.10-2.33, p ≤ 0.01).

Clinical presentation of NTS cases was comparable between serotypes (Table G in S1 Appendix). Rural residence (19%) and prehospital transfusion (21%) were remarkably more frequent in O5-antigen positive Typhimurium. Labored breathing was more frequent (43%) in O5-negative Typhimurium.

### Focal NTS infections

Osteo-articular infections were observed in twelve NTS cases. Eight of them had mono-arthritis: one was aspirated with isolation of NTS from the aspirate culture. Differential diagnosis of the other seven included both reactive and septic arthritis (6 knee, 1 shoulder). Four children (aged 4, 10, 10 & 26 months) presented dactylitis (differential diagnosis osteomyelitis), which affected both feet and both hands (n = 2) or a single hand (n = 2). In dactylitis, pus from incision & drainage was cultured in two children and NTS were isolated from both; two children had bone destruction on x-ray. Three children (1 mono-arthritis, 2 dactylitis) had a positive sickle cell screening test (Emmel test). Dactylitis with NTS isolation from pus was also observed in one child with negative blood cultures.

Cerebrospinal fluid was cultured in 11 children with NTS bloodstream infection with isolation of NTS in two of them. In addition, NTS was isolated from cerebrospinal fluid in a child with *Enterococcus faecium* bloodstream infection. Finally, NTS was isolated from urine in one NTS bloodstream infection and bilateral uveitis (not cultured) occurred in one NTS bloodstream infection.

### In-hospital clinical outcome and evolution

**Fig 4:**
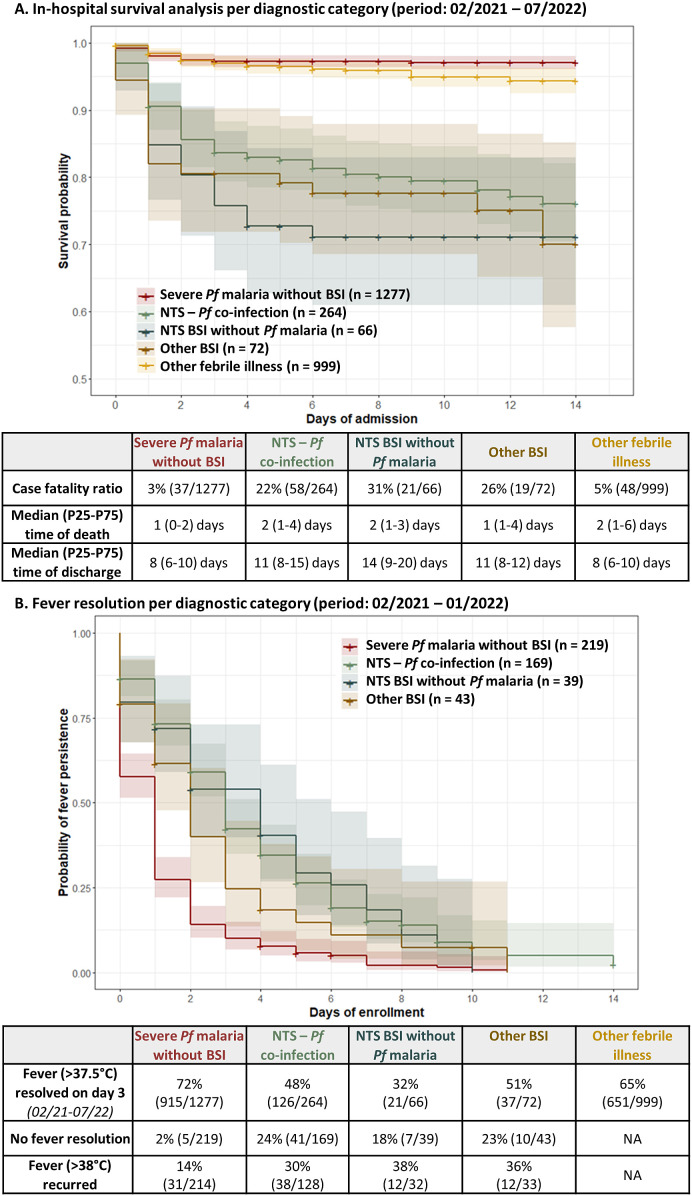
14-day hospital survival (A) & time-to-fever resolution (B) analysis per diagnostic category. The tables below the graph summarize additional categorical outcome data. Data on fever resolution were only available during the DeNTS study (02/2021-01/2022), unless otherwise specified. No fever resolution refers to children who left the hospital or died before fever resolution. Abbreviations: CFR: case fatality ratio, BSI: bloodstream infection, NTS: non-typhoidal *Salmonella*, *Pf*: *Plasmodium falciparum*, Day 3: day 3 after enrollment, NA: not applicable.

Overall case fatality was 7% (185/2682). A quarter of NTS cases died (24%, 80/331), whereas case fatality in severe *Pf* malaria without bloodstream infection was only 3% (37/1277; Fig 4A). Two-thirds (64%, 51/80) of NTS deaths occurred within 2 days of enrollment. Moreover, 25 eligible children with NTS bloodstream infection were not enrolled because they died before enrollment was possible (Fig 1). Among NTS cases, in-hospital case fatality tended to be higher in those without *Pf* malaria co-infection (22% vs 31%, p = 0.09). There was a non-significant difference in case-fatality between co-infections with recent *Pf* malaria (24%) versus co-infections with current *Pf* malaria (19%; p = 0.35; Table F in S1 Appendix). Case fatality of NTS bloodstream infections tended to be lower when caused by serotype Enteritidis (17%, 9/54) compared to Typhimurium (O5-antigen positive: 24% (39/163), O5-antigen negative: 29% (31/108), p = 0.15). Clinical signs and symptoms associated with death in NTS cases were mostly general signs of sepsis (Table 3).

**Table 3:** Sociodemographic factors and clinical signs and symptoms associated with death in non-typhoidal *Salmonella* (NTS) bloodstream infection.

	Fatal NTS cases (n = 80)	Non-fatal NTS cases (n = 251)	OR [95%CI]	P-value
**Female sex**	48.7% (n = 39)	47.8% (n = 120)	1.04 [0.63–1.72]	0.985
**Age under 2 years**	85.0% (n = 68)	73.3% (n = 184)	**2.06 [1.05–4.05]**	**0.047**
**Residence in rural village**	21.2% (n = 17)	12.3% (n = 31)	1.92 [1.00–3.68]	0.074
**Previous hospital admission** ***(in 12 months before fever onset)***	55.0% (n = 44)	54.4% (n = 136)	1.02 [0.62–1.70]	1
**>3 days of fever**	67.5% (n = 54)	54.5% (n = 137)	1.73 [1.02–2.94]	0.0566
**Cough**	37.5% (n = 30)	35.0% (n = 88)	1.11 [0.66–1.87]	0.793
**Vomiting**	21.2% (n = 17)	33.4% (n = 84)	0.54 [0.30–0.97]	0.054
**Diarrhea**	36.2% (n = 29)	30.2% (n = 76)	1.31 [0.77–2.22]	0.389
**Incapacity to eat/drink**	27.5% (n = 22)	5.57% (n = 14)	**6.42 [3.1–13.31]**	**<0.001**
**Prehospital antibiotics**	52.5% (n = 42)	41.4% (n = 104)	1.56 [0.94–2.59]	0.108
**Prehospital antimalarials**	52.5% (n = 42)	46.6% (n = 117)	1.27 [0.76–2.10]	0.43
**Prehospital transfusion**	22.5% (n = 18)	11.1% (n = 28)	**2.31 [1.20–4.45]**	**0.018**
**Prehospital iron supplements**	26.2% (n = 21)	27.4% (n = 69)	0.94 [0.53–1.66]	0.942
**Fever on enrollment**	46.2% (n = 37)	41.8% (n = 105)	1.2 [0.72–1.98]	0.572
**Tachycardia on enrollment**	81.2% (n = 65)	72.1% (n = 181)	1.68 [0.90–3.13]	0.138
**Tachypnea on enrollment**	75.0% (n = 60)	63.7% (n = 160)	1.71 [0.97–3.01]	0.085
**Hypoxia on enrollment**	26.2% (n = 21)	3.98% (n = 10)	**8.58 [3.83–19.2]**	**<0.001**
**Moderate acute malnutrition**	13.7% (n = 11)	19.9% (n = 50)	0.79 [0.38–1.64]*	0.654*
**Severe acute malnutrition**	26.2% (n = 21)	11.1% (n = 28)	2.70 [1.41–5.18]*	0.004*
**Prostration**	87.5% (n = 70)	56.9% (n = 143)	**5.29 [2.60–10.73]**	**<0.001**
**Clinical dehydration**	12.5% (n = 10)	6.37% (n = 16)	2.10 [0.91–4.83]	0.125
**Jaundice**	31.2% (n = 25)	6.77% (n = 17)	**6.26 [3.16–12.38]**	**<0.001**
**Not alert *(AVPU scale: V, P or U)***	38.7% (n = 31)	6.37% (n = 16)	**9.29 [4.72–18.29]**	**<0.001**
**Convulsions (before/on enrollment)**	30.0% (n = 24)	7.17% (n = 18)	**5.55 [2.82–10.92]**	**<0.001**
**Signs of meningitis *(bombing fontanel, neck stiffness or irritability)***	3.75% (n = 3)	3.58% (n = 9)	1.05 [0.28–3.97]	1
**Grunting**	37.5% (n = 30)	19.1% (n = 48)	**2.54 [1.46–4.40]**	**0.001**
**Labored breathing *(nasal flaring, chest/intercostal retractions)***	52.5% (n = 42)	24.3% (n = 61)	**3.44 [2.04–5.82]**	**<0.001**
**Hepatomegaly**	65.0% (n = 52)	31.8% (n = 80)	**3.97 [2.34–6.75]**	**<0.001**
**Splenomegaly**	58.7% (n = 47)	31.4% (n = 79)	**3.10 [1.85–5.21]**	**<0.001**
**Moderate anemia *(Hb <=9.3 g/dl)***	60.0% (n = 48)	70.9% (n = 178)	0.52 [0.30–0.91]*	0.032*
**Severe anemia *(Hb < 5 g/dl)***	6.25% (n = 5)	8.36% (n = 21)	0.46 [0.16–1.35]*	0.234*
**Hypoglycemia *(<45 mg/dl)***	28.7% (n = 23)	2.78% (n = 7)	**14.1 [5.75–34.4]**	**<0.001**
**Current *Pf* malaria**	21.5% (n = 17)	29.4% (n = 74)	0.66 [0.36–1.20]	0.216
**Recent *Pf* malaria**	51.8% (n = 41)	52.5% (n = 132)	0.97 [0.59–1.61]	1

*Pf: Plasmodium falciparum*, *reference: no acute malnutrition/no anemia

Figure 4B demonstrates that fever resolved slower in NTS bloodstream infection than in severe *Pf* malaria without bloodstream infection (HR_fever resolution_ = 0.37, 95%CI [0.30-0.46]). Three days after enrollment, fever had resolved in 45% (148/331) of NTS cases, compared to 72% in severe *Pf* malaria cases without bloodstream infection. A quarter (27%; 88/331) of NTS cases died or left the hospital before fever resolution. Hazards of fever resolution did not significantly differ in NTS with versus without *Pf* malaria co-infection (HR_fever resolution_ = 0.96, 95%CI [0.65-1.41]). After fever clearance, fever recurred in 31% (50/160) of NTS cases, which was significantly more than in severe *Pf* malaria without bloodstream infection (14% (31/214); OR=2.68, 95%CI [1.62-4.45]).

Control blood cultures were sampled in 159 NTS cases after 4–14 days of admission (median 8 days (P25-P75: 6–9 days)). From 26% of them (42/159), NTS were still isolated. Nine other control blood cultures revealed healthcare-associated bloodstream infections (3 *Acinetobacter* spp. from which 1 mixed with NTS, 2 *Enterobacter* spp., 1 *Pantoea dispersa*, 1 *E. coli*, 1 *Klebsiella* spp., 1 *S. aureus*). Two NTS cases had injection-related gluteal abscesses and five NTS cases had abscesses at (previous) peripheral catheter sites.

### Post-discharge outcome

**Fig 5:**
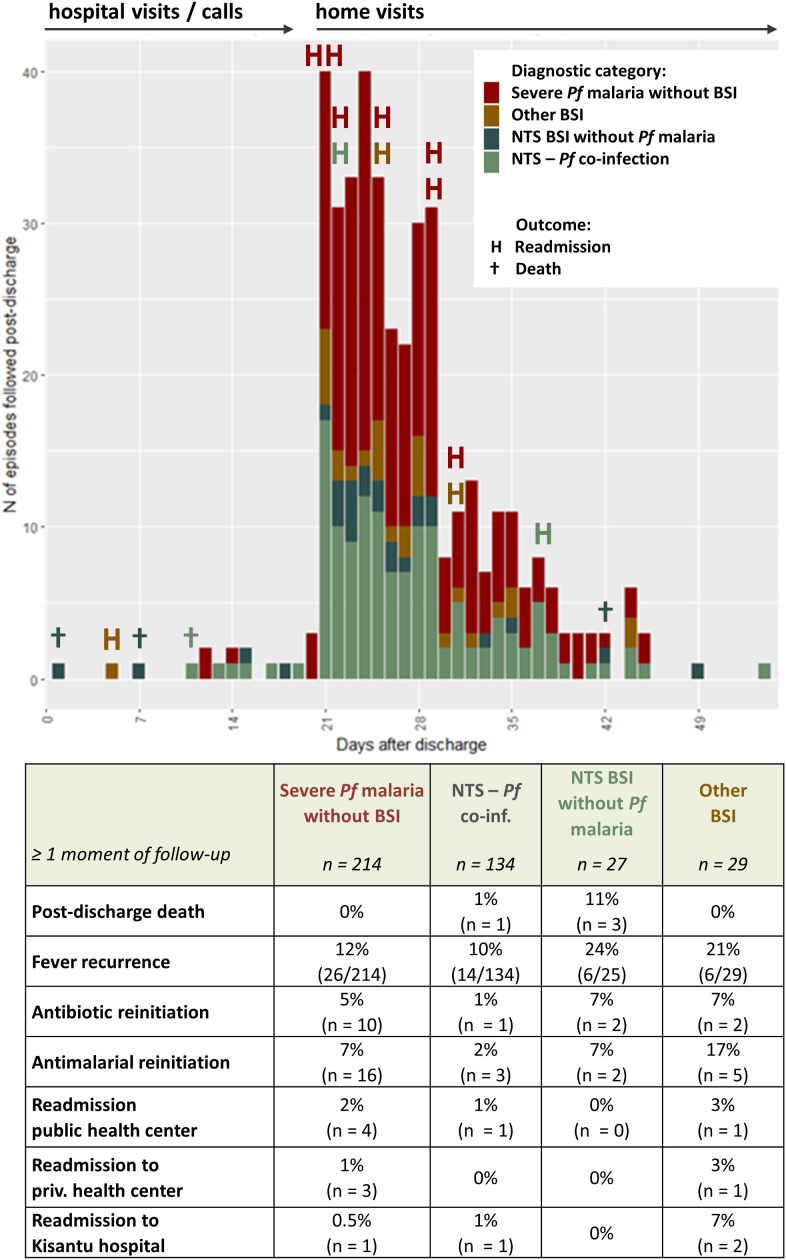
Duration of post-discharge follow-up and post-discharge outcome of non-typhoidal *Salmonella* (NTS) and other-than-NTS bloodstream infections (BSI) vs. age-matched controls with severe *Plasmodium falciparum (Pf)* malaria. Follow-up data originated from hospital visits or follow-up calls in the first 3 weeks after hospital discharge. All children followed ≥19 days after hospital discharge were visited at home. Each symbol represents one case of readmission to a health center/ Kisantu Hospital (H) or death (†), with the color of the symbol representing the diagnostic category of the case. Only data from the DeNTS study (February 2021-January 2022) presented. Due to logistic constraints, timing of follow-up can differ from the protocol. Timing of readmissions refers to the timing of readmission to the health center if children were readmitted to a health center first and then readmitted to Kisantu hospital.

From February 2021-January 2022 (DeNTS study), follow-up data from 49 post-discharge hospital visits, 231 phone calls and 391 home visits were collected. The median time of post-discharge follow-up was 26 days (P25-P75: 23–30 days). All children followed for ≥19 days post-discharge were visited at home (Fig 5). Of 163 NTS cases followed post-discharge, four (2.5%) died at 0, 7, 11 or 42 days post-discharge, while none of the malaria control cases died post-discharge. One of four NTS deaths was co-infected with recent *Pf* malaria, others were not co-infected. Fig B in S1 Appendix displays overall (in-hospital & post-discharge) survival per diagnostic category.

During post-discharge follow-up, fever recurrence was reported in 13% (20/159) of NTS survivors. Antibiotic and antimalarial re-initiation or readmission were rare (2%, 3% and 1%, respectively; Fig 5). However, after post-discharge follow-up, 33 children with NTS bloodstream infection presented again with severe febrile illness to Kisantu hospital and were re-enrolled (median interval: 99 days (P25-P75: 53–162 days)). Four of them had a second culture-confirmed bloodstream infection, including three caused by NTS.

## Discussion

### Main findings

Non-typhoidal *Salmonella* bloodstream infections occurred in one in eight enrolled children and 80% of NTS bloodstream infections were co-infected with *Pf* malaria. A third of children with recent *Pf* malaria had an NTS bloodstream infection. Other important risk factors for NTS were age < 2 years and malnutrition. The clinical presentation of NTS bloodstream infection was non-specific: fever >3 days, diarrhea or hepatosplenomegaly were often present, as were respiratory and neurological signs. General sepsis signs were associated with in-hospital death of NTS cases. Non-typhoidal *Salmonella* co-infected 6% of severe *Pf* malaria cases and the case fatality ratio in children with NTS bloodstream infection (24%) was substantially higher than in severe *Pf* malaria without bloodstream infection (3%). Delayed fever resolution, slow blood culture clearance, focal infections and post-discharge deaths were observed in NTS cases.

### Strengths & limitations

This is the largest and most comprehensive prospective, observational study presenting clinical data on invasive NTS infections in sub-Saharan African children (Table I in S1 Appendix). Integration in blood culture surveillance enabled high enrollment rates and provided data on deaths before enrollment. Unlike most surveillance studies, data quality was assured through standardized clinical data collection by a dedicated and well-trained research team, microbiological quality control and assurance, and rigorous data monitoring. Nevertheless, the hospital-based study design also created bias due to health care seeking behavior and access (which might have been affected by the COVID-pandemic), due to selection of most severely ill patients, and due to delays in hospital presentation, blood culture sampling and enrollment. Secondly, the suboptimal sensitivity of blood cultures, particularly for *Streptococcus pneumoniae* in a context of frequent prehospital antibiotic use, inherently biased the diagnostic categorization in this study. Furthermore, limited routine laboratory capacity/use restricted diagnosis of HIV and sickle cell disease (sickle cell disease known in only 0.5% of children in this study versus ±2% of children born in DR Congo) [31] and required a modified, possibly less strict, severe *Pf* malaria definition. Furthermore, the group with NTS-*Pf* malaria co-infection might be relatively heterogenous, as we did not account for severity (severe versus uncomplicated) and stage of *Pf* malaria infection (current versus recent). Typhoid conjugate vaccination in the last 6 study months may have impacted healthcare seeking and NTS epidemiology, although cross-protection for NTS seems limited [32]. Finally, once or twice daily temperature measurement combined with subjective fever reporting may have been insufficiently granular to assess defervescence.

### *Pf* malaria, particularly recent *Pf* malaria, was the most important risk factor for NTS

Four out of five bloodstream infections in this cohort were caused by NTS, compared with approximately one in three community-acquired bloodstream infections in children elsewhere in sub-Saharan Africa.[33,34] The high NTS occurrence likely reflects the intense year-round *Pf* malaria transmission in this rural setting, which peaks during the rainy season, in the country with the second highest and non-declining incidence rate in the world. This hypothesis is consistent with the high proportion of *Pf* malaria co-infections in this cohort. [6,10] Half of NTS bloodstream infections were co-infected with recent *Pf* malaria and a quarter with current *Pf* malaria. Children with recent *Pf* malaria are predisposed to NTS infection (in contrast to other bacterial pathogens) due to prolonged immunosuppression after malarial hemolytic anemia promoting intracellular NTS survival [24,35,36]. This susceptibility is mediated by heme-related pathways favoring the intracellular niche of NTS growth: hemozoin, a heme degradation product, impairs macrophage activation, induction of heme oxygenase-1, a heme degrading enzyme, inhibits the neutrophilic oxidative burst, and heme itself is a source of iron which promotes replication of the siderophilic NTS. These heme-mediated immunosuppressing effects persist for weeks after acute malaria and take longer to recuperate than hemoglobin levels, which may partially explain the absent association between NTS and anemia. [24,37] The accumulation of long-lasting immunosuppressing effects from recurrent malaria may explain the observed increase in NTS cases and case fatality in the late rainy season [10]. Co-infections of NTS with current *Pf* malaria have been related to increased gut permeability due to parasite sequestration and L-arginine deficiency, complement consumption, splenic and humoral immunity dysfunction, and impaired cellular immunity due to cytokine dysregulation [24]. Children with an NTS infection without *Pf* co-infection were almost all younger than 2 years, which is the approximate age required for mature CD4+ T-cell immune responses and antibody-dependent serum bactericidal activity against NTS [36,38–46]. Children <6 months are probably less exposed and benefit from passive immunity via breastfeeding [47]. Acute malnutrition was frequent in NTS irrespective of malaria status. Malnutrition may have further impaired immunity, as observed in its association with fatal NTS [8]. Often, risk factors co-occur and their immunological effects may interact. Differences in pathways and extent of impaired immunity may partially explain the stepwise increase in case fatality from NTS co-infections with current *Pf* (19%), to NTS co-infections with recent *Pf* (24%), to NTS without *Pf* co-infection (31%). Current *Pf* malaria may cause a more transient innate, humoral and cellular immune impairment that facilitates infection, whereas the prolonged hemolysis-mediated immune dysregulation of recent *Pf* malaria promotes intracellular NTS survival and impairs bacterial clearance. Children without *Pf* co-infection may have been intrinsically more susceptible, e.g., due to younger age or (underreported/non-diagnosed) sickle cell disease. Nevertheless, case fatality is likely driven more by timeliness of hospital admission and illness severity at presentation. Consistent with previous data [15], we observed differences in prehospital care, with longer fever duration before admission and more frequent prehospital administration of antibiotics, antimalarials, and blood transfusions among children with NTS and recent or no *Pf* malaria co-infection compared to those with a current *Pf* malaria co-infection. Earlier recognition of danger signs in children with NTS - *Pf* malaria co-infections, facilitated by malaria and anemia diagnostics, may have expedited their referral and access to treatment.

### NTS bloodstream infections present as severe multisystemic febrile illness, like severe *Pf* malaria

In analogy to previous smaller/retrospective studies on the clinical presentation of NTS, this study could not identify clinical signs and symptoms that were specific for NTS bloodstream infections. Table I in S1 Appendix summarizes previous studies on clinical presentation of invasive NTS infections [36,39–46,48–61]. As a result of their multisystemic non-specific presentation and malaria co-infections, the possible diagnosis of NTS bloodstream infections is easily overlooked. Moreover, the clinical criteria to start antibiotics described in the WHO Pocketbook of hospital care for children fail to detect 60% of invasive NTS infections [3] and rapid diagnostic tests to rule out bacterial infections in hospital-admitted children are not yet available for sub-Saharan Africa [62–66]. To avoid missed NTS cases, a high index of suspicion for NTS in *Pf* malaria and NTS-endemic settings is required, also in children already diagnosed as severe *Pf* malaria, and particularly in children with general sepsis/danger signs such as prostration, incapacity to eat/drink and hypoglycemia, which were in the present cohort associated with fatal NTS. Based on the clinical presentation data from this study, we recently developed a clinical prediction model to estimate the risk of NTS bloodstream infection in children presenting with severe febrile illness to the hospital [67]. After external validation, this model might help clinicians to modify empirical antibiotics to improve coverage of NTS bloodstream infections on hospital arrival [67]. Nevertheless, blood cultures remain necessary for diagnostic confirmation and antibiotic susceptibility testing and access to blood culture diagnostics must urgently be upscaled in sub-Saharan Africa [68,69].

Focal infections (osteo-articular infections, meningitis) should trigger NTS suspicion, and, *vice versa*, NTS isolation should trigger active screening for focal infections. Vaso-occlusive crisis in young children with sickle cell disease frequently present with dactylitis (hand-foot syndrome) [70,71]. The association between NTS and dactylitis may reflect bone infarcts favoring osteomyelitis with NTS circulating in blood or dormant NTS in bone marrow tissues [70]. Hemolytic anemia and parasite sequestration in *Pf* malaria might mimic sickle-cell mediated vaso-occlusion. The observed arthritis and uveitis/endophthalmitis could be septic or reactive and merit microbiological culturing and appropriate surgical/medical care [72–75]. Recognition of focal infections is also important, as they require prolonged duration of antibiotic treatment [13].

### Poor survival mandates broad differential diagnosis, improved sepsis care and effective antibiotics

In-hospital case fatality of NTS bloodstream infection was almost ten times higher than case fatality of severe *Pf* malaria without bloodstream infection (24% versus 3%). The latter was lower than reported in severe *Pf* malaria studies, probably due to underdiagnosis of bacterial co-infections in these studies [76]. Furthermore, deaths mostly occurred in the first 2 days of admission and an additional 25 NTS deaths occurred even prior to enrollment.

The large impact on malaria survival of co-infections and earliness of in-hospital death stresses that severe febrile illness merits a comprehensive assessment that does not focus on a single disease etiology (*i.e.,* at least including blood cultures and malaria diagnostics) [2]. Given the high and early case fatality, universal broad-spectrum empirical antibiotic coverage for children with severe febrile illness in sub-Saharan African hospitals can be justified. This is supported by the low number needed to treat per culture-confirmed bloodstream infection (seven). Blood cultures sampled at admission, along with fever and clinical evolution, can help to stop/target antibiotics after 72 hours. Interestingly, most blood cultures from which NTS was isolated showed signs of growth within 2 days of incubation. In addition, the differential timing of fever resolution in severe *Pf* malaria versus NTS bloodstream infections (72% versus 45% fever resolution within 3 days) can be informative for diagnosis.

In many sub-Saharan African hospitals, deaths mostly occur early during hospital admission, which has been linked to poor danger sign recognition and poor adherence to sepsis care guidelines [77–79]. Also in our study, fatal cases with NTS bloodstream infection presented with sepsis-like danger signs and symptoms, which stresses the need to train (frontline) healthcare workers in danger sign recognition and to accelerate hospital referral, as discussed elsewhere [15].

Case fatality of NTS was comparable to survival in the 20^th^ century, but higher than studies from the last 20 years (Table I in S1 Appendix) [39,40,42–46,48–55,57–61]. This might be explained by the fact that three-quarters of isolated NTS were resistant to third generation cephalosporins and fluoroquinolones, which were the most frequently used antibiotics to treat NTS bloodstream infections in the first days of admission. This is discussed more in depth in the manuscript reporting antibiotic susceptibility and treatment data from the TreNTS study (data to be published. Suboptimal treatment may also have contributed to delayed fever resolution, fever recurrence, slow blood culture clearance, long hospital stays and post-discharge deaths in NTS cases, although host (impaired immunity), pathogen (intracellular persistence) and environmental risk factors (water, sanitation and hygiene) might also have contributed [8].

### NTS control implies malaria control and O5-antigen-independent NTS vaccines for infants

The high case fatality and difficulties to diagnose NTS bloodstream infections mandates better NTS control. The frequent NTS-*Pf* malaria co-infections highlight the importance of *Pf* malaria control. In other sub-Saharan African countries, successful malaria control programs have reduced the NTS burden. Now that the RTS,S/AS01 (and soon R21/Matrix-M) malaria vaccine is piloted in routine childhood immunizations, its impact on the NTS burden must be assessed [80,81]. Other current public health interventions which can reduce NTS are childhood nutrition and water, sanitation and hygiene interventions. Vaccines against NTS are being developed, but should, as previously observed, also cover O5-antigen negative Typhimurium [25,82]. Finally, the observed young age of NTS cases illustrates that immunization must start at infant age [83,84].

## Conclusion

Non-typhoidal *Salmonella* bloodstream infections were frequent. Recent *Pf* malaria was a major risk factor for NTS bloodstream infection. NTS also co-infected children with severe *Pf* malaria and NTS bloodstream infection could not be differentiated from other severe febrile illness etiologies on a clinical basis. A fourth of children with NTS bloodstream infections died in-hospital, mainly within 2 days of admission. General danger/sepsis signs were associated with NTS case fatality. To reduce NTS deaths, a high index of suspicion for NTS with low threshold to initiate empirical antibiotics irrespective of presumed diagnosis on admission and early danger sign recognition and sepsis care are warranted.

## Supporting information

S1 Appendix Supporting information for manuscript. **Table A in S1 Appendix.** Criteria defining suspected bloodstream infection. If children fulfilled both criteria when they arrived at the hospital, bloodstream infection was suspected, and a blood culture was sampled and worked up.**Table B in S1 Appendix.** Actions taken to control and assure quality of the study.**Table C in S1 Appendix.** Comparison of WHO definition of severe *Pf* malaria [12] versus the severe *Pf* malaria definition applied in this study. Adaptations were made because it was not feasible (due to unavailability of radiological or biochemical tests, complicated clinical evaluation or insufficiently granular follow-up) to measure all WHO criteria in Kisantu hospital. **Table D in S1 Appendix.** Cumulative antibiogram (% susceptible) of pathogens isolated from the blood culture sampled on admission for which reference antibiotic susceptibility testing was performed.**Fig A in S1 Appendix.** Comparison of hemoglobin levels and *Plasmodium falciparum (Pf)* parasite density across diagnostic categories.**Table E in S1 Appendix.** Comparative clinical presentation of children with severe *Plasmodium falciparum* (*Pf*) malaria without bloodstream infection (BSI), non-typhoidal *Salmonella* (NTS) bloodstream infection or NTS – *Pf* malaria co-infection.**Table F in S1 Appendix.** Comparative clinical presentation of children with non-typhoidal *Salmonella* (NTS) bloodstream infection (BSI) according to current, recent, or no *Plasmodium falciparum* (*Pf*) malaria co-infection.**Table G in S1 Appendix.** Comparison of clinical presentation of children with non-typhoidal Salmonella (NTS) bloodstream infection (BSI) according to serotype.**Table H in S1 Appendix.** Case fatality and timing of death according to presence of culture-confirmed bloodstream infection (BSI) and malaria status.**Fig B in S1 Appendix.** Overall (in-hospital and post-discharge) survival analysis according to diagnostic strata.**Fig C in S1 Appendix**. Seasonal distribution of cases and deaths per diagnostic category.**Fig D in S1 Appendix.** Seasonal distribution of NTS cases according to serotype.**Table I in S1 Appendix.** Systematic literature review of studies presenting data on clinical presentation of children with non-typhoidal *Salmonella* (NTS) bloodstream infection in sub-Saharan Africa [39,41,42,44–46,48–56,61]. The PubMed search string was: *Salmonella* [TIAB] AND (non-typh*[TIAB] OR nontyph*[TIAB] OR typhimurium[TIAB] OR enteritidis[TIAB]) AND (bacterem*[TIAB] OR bacteraem*[TIAB] OR “blood culture”[TIAB] OR “bloodstream infection”[TIAB] OR sepsis [TIAB] OR invasive [TIAB])). Systematic review of studies presenting treatment efficacy data for NTS bloodstream infection was previously published by Tack et al. in BMC Medicine in 2020.
(DOCX)
