# Supplementary material for: Non-typhoidal Salmonella co-infect and complicate Plasmodium falciparum malaria in children under-five: A prospective cohort study on clinical presentation and outcome in Kisantu district hospital, DR Congo
Source: PLoS Negl Trop Dis. 2026 Jun 24;20(6):e0014457. doi: 10.1371/journal.pntd.0014457 (PMC13327514; doi:10.1371/journal.pntd.0014457)
Supplement: S1 Appendix — Table A in S1 Appendix. Criteria defining suspected bloodstream infection. If children fulfilled both criteria when they arrived at the hospital, bloodstream infection was suspected, and a blood culture was sampled and worked up.Table B in S1 Appendix. Actions taken to control and assure quality of the study.Table C in S1 Appendix. Comparison of WHO definition of severe Pf malaria [12] versus the severe Pf malaria definition applied in this study. Adaptations were made because it was not feasible (due to unavailability of radiological or biochemical tests, complicated clinical evaluation or insufficiently granular follow-up) to measure all WHO criteria in Kisantu hospital. Table D in S1 Appendix. Cumulative antibiogram (% susceptible) of pathogens isolated from the blood culture sampled on admission for which reference antibiotic susceptibility testing was performed.Fig A in S1 Appendix. Comparison of hemoglobin levels and Plasmodium falciparum (Pf) parasite density across diagnostic categories.Table E in S1 Appendix. Comparative clinical presentation of children with severe Plasmodium falciparum (Pf) malaria without bloodstream infection (BSI), non-typhoidal Salmonella (NTS) bloodstream infection or NTS – Pf malaria co-infection.Table F in S1 Appendix. Comparative clinical presentation of children with non-typhoidal Salmonella (NTS) bloodstream infection (BSI) according to current, recent, or no Plasmodium falciparum (Pf) malaria co-infection.Table G in S1 Appendix. Comparison of clinical presentation of children with non-typhoidal Salmonella (NTS) bloodstream infection (BSI) according to serotype.Table H in S1 Appendix. Case fatality and timing of death according to presence of culture-confirmed bloodstream infection (BSI) and malaria status.Fig B in S1 Appendix. Overall (in-hospital and post-discharge) survival analysis according to diagnostic strata.Fig C in S1 Appendix. Seasonal distribution of cases and deaths per diagnostic category.Fig D in S1 Appendix. Seas [file pntd.0014457.s001.docx]

**Supporting information for manuscript:
“Non-typhoidal *Salmonella* co-infect and complicate *Plasmodium falciparum* malaria in children under-five: a prospective cohort study on clinical presentation and outcome in Kisantu district hospital, DR Congo”**

Bieke Tack ^1,2,3,*^, Daniel Vita ^4^, Jules Mbuyamba ^5,6^, José Nketo ^7^, Emmanuel Ntangu ^4^, Marie-France Phoba ^5,6^, Aimée Luyindula ^4^, Gaëlle Nkoji ^5,8^, Hornela Vuvu ^4^, Anne-Sophie Heroes ^1,2^, Justin Im ^9,10^, Birkneh Tilahun Tadesse ^9^, Mohamadou Siribie ^9^, Hyon Jin Jeon ^9,11^, Florian Marks ^9,11,12,13^, Liselotte Hardy ^1^, Erika Vlieghe ^1,14,15^, Jaan Toelen ^3,16^, Jan Jacobs ^1,2,#^, Octavie Lunguya ^5,6,#^

**Affiliations:**

1. Department of Clinical Sciences, Institute of Tropical Medicine, Antwerp, Belgium
2. Department of Microbiology, Immunology and Transplantation, KU Leuven, Belgium
3. Department of Pediatrics, University Hospitals Leuven, Belgium
4. Saint Luc Hôpital Général de Référence Kisantu, Democratic Republic of Congo
5. Department of Microbiology, Institut National de Recherche Biomédicale, Kinshasa, Democratic Republic of Congo
6. Department of Medical Biology, University Teaching Hospital of Kinshasa, Democratic Republic of Congo
7. Zone de Santé Kisantu, Kisantu, Democratic Republic of Congo
8. Faculty of Medicine, Université Protestante au Congo, Democratic Republic of Congo
9. International Vaccine Institute, Seoul, Republic of Korea
10. Research Investment for Global Health Technology (RIGHT) Foundation, Seoul, Republic of Korea
11. Madagascar Institute for Vaccine Research, University of Antananarivo, Antananarivo, Madagascar
12. Cambridge Institute of Therapeutic Immunology and Infectious Disease, University of Cambridge School of Clinical Medicine, Cambridge, UK
13. Heidelberg Institute of Global Health, University of Heidelberg, Heidelberg, Germany
14. Faculty of Medicine and Health Sciences, University of Antwerp, Belgium
15. Department of General Internal Medicine Infectious Diseases and Tropical Medicine, University Hospital Antwerp, Belgium
16. Department of Development and Regeneration, KU Leuven, Leuven, Belgium

* Corresponding author: Bieke Tack, [btack@itg.be](mailto:btack@itg.be)
# Equally contributed

**Table A.** Criteria defining suspected bloodstream infection. If children fulfilled both criteria when they arrived at the hospital, bloodstream infection was suspected, and a blood culture was sampled and worked up.

| **CRITERIUM 1: fever or hypothermia** | | | |
| --- | --- | --- | --- |
|  | Rectal | Axillary | Tympanic |
| Fever upon presentation | > 38.0 °C | > 37.5°C | > 37.5°C |
| Hypothermia upon presentation | < 36.0 °C | ≤ 35.5°C | ≤ 35.5°C |
| Subjective fever in the past 48 hours (as reported by the caretaker) | | | |
| **CRITERIUM 2: Minimum 1 severity sign present** | | | |
| Quick SOFA score ≥ 1 | Hypotension | | |
|  | Confusion | | |
|  | Increased respiratory rate | | |
| Suspicion of severe localized infection | Pneumonia | | |
|  | Meningitis | | |
|  | Osteomyelitis | | |
|  | Complicated urinary tract infection | | |
|  | Abscess | | |
|  | Severe skin or soft tissue infection | | |
|  | Abdominal infection | | |
| Suspicion of other severe systemic infection | Severe malaria | | |
|  | Typhoid fever | | |

| **Table B.** Actions taken to control and assure quality of the study. | |
| --- | --- |
|  | **Quality assurance and control actions** |
| **Study supervision, monitoring & coordination** | - The principal investigator (BT) organized on-site, hands-on training of the research team 2 weeks prior to first enrollment. - The principal investigator (BT) provided on-site, hands-on supervision of the first 6 weeks of enrollment. - Further supervision and coordination were organized via weekly virtual meetings and regular site visits. |
| **Data management** | - Data collection in electronic case report forms (RedCap, Vanderbilt University, Tennessee, US) - Data verification by an on-site data monitor and monitored remotely. |
| **Standardization of clinical data collection** | - Data collection by dedicated and trained research team - Clinical data collection according to standardized operating procedures. - Use of per protocol defined study definitions. |
| **Malaria microscopy** | - Malaria microscopy refresher trainings were organized for the hospital laboratory staff. - Monthly random quality control of 3 slides to evaluate positivity, species identification, parasite density determination and quality of Giemsa staining. - All malaria microscopy slides were stored for quality control and additional reading was organized in case of discordances between malaria microscopy and malaria rapid antigen tests was organized: second reading by local expert, third reading if necessary, by expert at Institute of Tropical Medicine (ITM, Antwerp, Belgium). |
| **Blood culture diagnostics** | - On-site, blood cultures were sampled and worked up according to standardized operating procedures that follow international laboratory standards, including quality control steps. This work-up (manual blood culture incubation, biochemical identification, disk diffusion for antibiotic susceptibility testing) has been described in previous blood culture studies [1–4]. - Bacterial isolates were sent on tryptic soy agar to ITM for reference testing. At ITM, bacterial identification was confirmed with MALDI-TOF MS (Bruker, Billerica, USA) and NTS were serotyped with commercial antisera (Pro-lab Diagnostics, Richmond Hill, Canada and Sifin, Berlin, Germany). Reference testing of antibiotic susceptibility testing was performed with the Sensititre AST System (Sensititre Nephelometer, AIM automated inoculator & Vizion digital MIC viewing system; Thermo Fisher, Waltham, USA) with customized dry Sensititre plates, in addition to azithromycin, pefloxacin and ciprofloxacin disk diffusion testing (Rosco, Taastrup, Denmark). Procedures followed during reference testing can be retrieved at <https://labhub.itg.be/> . Interpretative criteria for antibiotic susceptibility testing were CLSI M100 Edition 33 [5]. |

| **Table C.** Comparison of WHO definition of severe *Pf* malaria [6] versus the severe *Pf* malaria definition applied in this study. Adaptations were made because it was not feasible (due to unavailability of radiological or biochemical tests, complicated clinical evaluation or insufficiently granular follow-up) to measure all WHO criteria in Kisantu hospital. | |
| --- | --- |
| **Original WHO criteria for severe *Pf* malaria** | **Criteria for severe *Pf* malaria used in this study** |
| Impaired consciousness:  Blantyre coma score < 3 in children | AVPU scale: reaction to voice, reaction to pain or unresponsive |
| Prostration | |
| Multiple convulsions: >2 episodes within 24h | Convulsions reported by caretaker or observed on admission |
| Acidosis: A base deficit of >8 mEq/L or, if not available, a plasma bicarbonate level of <15 mmol/L or venous plasma lactate ≥ 5 mmol/L. Severe acidosis manifests clinically as respiratory distress (rapid, deep, labored breathing). | Fast breathing according to age (WHO pocketbook for hospital care of children)  or  Respiratory rate >30/min with oxygen saturation <92%  or  Grunting  or  Labored breathing (nasal flaring, chest/intercostal retractions) |
| Pulmonary oedema: Radiologically confirmed or oxygen saturation <92% on room air with a respiratory rate >30/min, often with chest indrawing and crepitations on auscultation |  |
| Jaundice: Plasma or serum bilirubin > 3 mg/dl with a parasite count >100,000/μL | Clinical icterus with a parasite count >100,000/μL |
| Renal impairment: Plasma or serum creatinine >3 mg/dL or blood urea >20 mmol/L | No renal impairment evaluated |
| Severe malarial anemia:  Hemoglobin concentration ≤ 5 g/dL or a hematocrit of ≤ 15% with a parasite count > 10,000/μL | |
| Hypoglycemia: Blood or plasma glucose <40 mg/dL | |
| Significant bleeding: Including recurrent or prolonged bleeding from the nose, gums or venipuncture sites; hematemesis or melaena | Not evaluated |
| Shock: Compensated shock is defined as capillary refill ≥3 s or temperature gradient on leg (mid to proximal limb), but no hypotension. Decompensated shock is defined as systolic blood pressure <70 mm Hg in children or <80 mmHg in adults, with evidence of impaired perfusion (cool peripheries or prolonged capillary refill). | Capillary refill ≥3 s with tachycardia according to age (WHO pocketbook for hospital care of children)  or  unmeasurable oxygen saturation due to poor perfusion |
| Hyperparasitemia: *Pf* parasitemia > 10% | *Pf* parasitemia > 500,000/µL |

| \| **Table D.** Cumulative antibiogram (% susceptible) of pathogens isolated from the blood culture sampled on admission for which reference antibiotic susceptibility testing was performed. \| \| \| \| \| \| \| \| \| \| \| \| \| \| \| \| \| \| --- \| --- \| --- \| --- \| --- \| --- \| --- \| --- \| --- \| --- \| --- \| --- \| --- \| --- \| --- \| --- \| --- \| \|  \| **Penicillin** \| **Methicillin** \| **Ampicillin** \| **Amoxicillin + clavulanic acid** \| **Ceftriaxone** \| **Ceftazidime** \| **Piperacillin + tazobactam** \| **Meropenem** \| **Gentamicin** \| **Amikacin** \| **Ciprofloxacin** \| **Cotrimoxazole** \| **Chloramphenicol** \| **Azithromycin** \| **Vancomycin** \| **Doxycycline** \| \| **Non-typhoidal *Salmonella* (n = 325)** \|  \|  \| 9% \|  \| 26% \| 26% \| 63% \| 100% \| ER \| ER \| 28% \| 14% \| 10% \| 98% \| ER \|  \| \| ***Klebsiella* spp. (n = 21)** *(17 K. pneumoniae, 3 K. varriicola* *,  1 K. aerogenes)* \| ER \| ER \| ER \| 67% \| 5% \| 5% \| 86% \| 100% \| 14% \| 95% \| 52% \| 10% \|  \|  \| ER \| 43% \| \| ***Escherichia coli* (n = 11)** \|  \|  \| 0% \| 82% \| 27% \| 45% \| 91% \| 100% \| 55% \| 100% \| 73% \| 9% \|  \|  \| ER \| 100% \| \| ***Enterobacter* spp. (n = 9)**  *(8 E. cloacae complex, 1 E. bugandensis)* \| ER \| ER \| ER \| ER \| 56% \| 56% \| 100% \| 100% \| 56% \| 100% \| 56% \| 56% \|  \|  \| ER \| 44% \| \| ***Salmonella* Typhi (n = 5)** \|  \|  \| 0% \|  \| 100% \| 100% \| 100% \| 100% \| ER \| ER \| 80% \| 0% \| 100% \| 100% \| ER \|  \| \| ***Acinetobacter* spp*.* (n = 3)**  *(A. baumanni, A. nosocomialis, A. variabilis)* \| ER \| ER \| ER \| ER \| ER \| 33% \| 33% \| 33% \| 0% \| 0% \| 0% \| ER \|  \|  \| ER \| 33% \| \| ***Citrobacter koseri* (n = 2)** \| ER \| ER \| ER \| 100% \| 100% \| 100% \| 100% \| 100% \| 100% \| 100% \| 100% \| 100% \|  \|  \| ER \| 100% \| \| ***Stenotrophomonas maltophilia* (n = 1)** \| ER \| ER \| ER \| ER \| ER \| 0% \| ER \| ER \| ER \| ER \|  \| 100% \|  \|  \| ER \|  \| \| ***Leclercia adecarboxylata* (n = 1)** \|  \|  \| 100% \| 100% \| 100% \| 100% \| 100% \| 100% \| 100% \| 100% \| 0% \| 0% \|  \|  \| ER \| 100% \| \| ***Staphylococcus aureus* (n = 12)** \| 25% \| 50% \|  \|  \|  \|  \|  \|  \| 67% \| 100% \| 75% \| 83% \|  \|  \| 100% \| 92% \| \| ***Enterococcus faecium* (n = 1)** \| ER \| ER \| 0% \|  \|  \|  \|  \|  \| 100% \|  \|  \|  \|  \|  \| 100% \|  \| \| *ER: Expected resistance according to EUCAST Expected Resistant Phenotypes version 1.2*[7] *or expected in vivo resistance according to CLSI M100 [5] . No data (grey shade) are presented if the specific antibiotic was not tested or if no interpretative criteria were available. Antibiotic names are colored according to their AWaRe classification (green = Access antibiotics, yellow = Watch antibiotics). Resistance percentages are shaded in green if >95% was susceptible and in red if <30% was susceptible* [8,9]*.* \| \| \| \| \| \| \| \| \| \| \| \| \| \| \| \| \| |
| --- | --- | --- | --- | --- | --- | --- | --- | --- | --- | --- | --- | --- | --- | --- | --- | --- | --- | --- | --- | --- | --- | --- | --- | --- | --- | --- | --- | --- | --- | --- | --- | --- | --- | --- | --- | --- | --- | --- | --- | --- | --- | --- | --- | --- | --- | --- | --- | --- | --- | --- | --- | --- | --- | --- | --- | --- | --- | --- | --- | --- | --- | --- | --- | --- | --- | --- | --- | --- | --- | --- | --- | --- | --- | --- | --- | --- | --- | --- | --- | --- | --- | --- | --- | --- | --- | --- | --- | --- | --- | --- | --- | --- | --- | --- | --- | --- | --- | --- | --- | --- | --- | --- | --- | --- | --- | --- | --- | --- | --- | --- | --- | --- | --- | --- | --- | --- | --- | --- | --- | --- | --- | --- | --- | --- | --- | --- | --- | --- | --- | --- | --- | --- | --- | --- | --- | --- | --- | --- | --- | --- | --- | --- | --- | --- | --- | --- | --- | --- | --- | --- | --- | --- | --- | --- | --- | --- | --- | --- | --- | --- | --- | --- | --- | --- | --- | --- | --- | --- | --- | --- | --- | --- | --- | --- | --- | --- | --- | --- | --- | --- | --- | --- | --- | --- | --- | --- | --- | --- | --- | --- | --- | --- | --- | --- | --- | --- | --- | --- | --- | --- | --- | --- | --- | --- | --- | --- | --- | --- | --- | --- | --- | --- | --- | --- | --- | --- | --- | --- | --- | --- | --- | --- | --- | --- | --- | --- | --- | --- | --- | --- | --- | --- | --- | --- | --- | --- | --- | --- |

| **Fig A.** Comparison of hemoglobin levels and *Plasmodium falciparum (Pf)* parasite density across diagnostic categories. |
| --- |
| 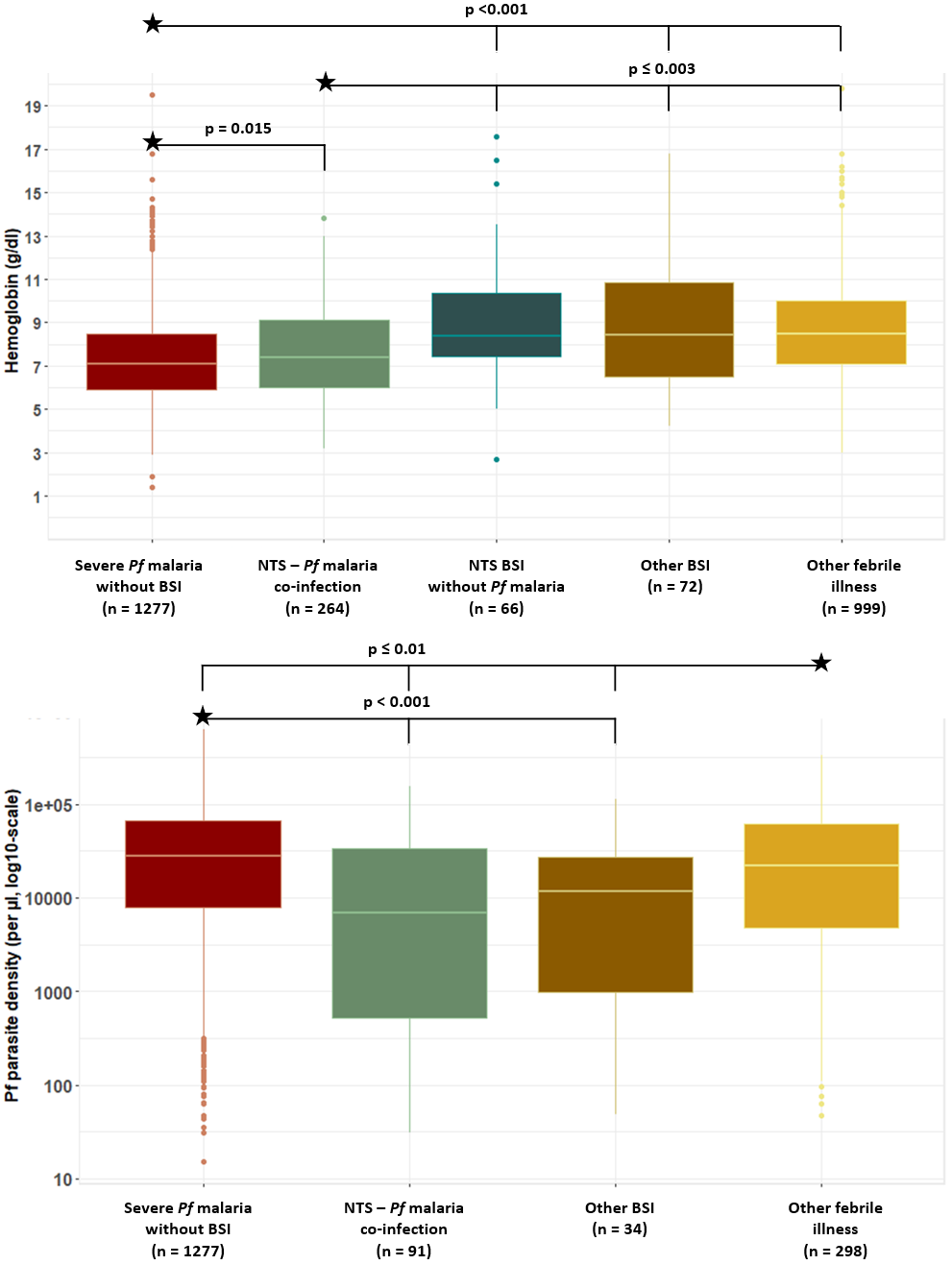 |
| *Statistical significance testing was performed with the unpaired Wilcoxon log-rank test. P-values apply to the difference between the category marked with a star and each category indicated by a short vertical drop down line. Abbreviations: NTS: non-typhoidal Salmonella, BSI: bloodstream infection, Pf: Plasmodium falciparum* |

| \| **Table E.** Comparative clinical presentation of children with severe *Plasmodium falciparum* (*Pf*) malaria without bloodstream infection (BSI), non-typhoidal *Salmonella* (NTS) bloodstream infection or NTS – *Pf* malaria co-infection. \| \| \| \| \| \| \| \| \| \| \| --- \| --- \| --- \| --- \| --- \| --- \| --- \| --- \| --- \| --- \| \|  \| **Severe *Pf* malaria without BSI**  (n = 1277) \| **NTS - *Pf* malaria**  **co-infection**  (n = 264) \| **% in NTS BSI**  **without *Pf* malaria** (n = 66) \| **NTS – *Pf* malaria**  **co-infection**  **vs. severe *Pf* malaria**  **without BSI** \| \| **NTS BSI**  **without *Pf* malaria**  **vs. severe *Pf* malaria**  **without BSI** \| \| **NTS - *Pf* malaria**  **co-infection**  **vs. NTS BSI**  **without *Pf* malaria** \| \| \| OR [95% CI] \| p-value \| OR [95% CI] \| p-value \| OR [95% CI] \| p-value \| \| **Female sex** \| 48.6% (n = 621) \| 51.1% (n = 135) \| 34.8% (n = 23) \| 1.11 [0.85 - 1.44] \| 0.500 \| 0.57 [0.34 - 0.95] \| 0.039 \| 1.96 [1.12 - 3.43] \| 0.026 \| \| **Age under 2 years** \| 59.2% (n = 756) \| 71.5% (n = 189) \| 93.9% (n = 62) \| **1.74 [1.30 - 2.32]** \| **<0.001** \| **10.7 [3.86 - 29.5]** \| **<0.001** \| **0.16 [0.06 - 0.46]** \| **<0.001** \| \| **Residence in rural village** \| 8.8% (n = 113) \| 14.0% (n = 37) \| 16.6% (n = 11) \| **1.68 [1.13 - 2.50]** \| **0.014** \| 2.06 [1.05 - 4.05] \| 0.055 \| 0.81 [0.39 - 1.70] \| 0.725 \| \| **Previous hospital admission** *(in 12 months before fever onset)* \| 46.7% (n = 596) \| 55.5% (n = 146) \| 51.5% (n = 34) \| **1.42 [1.09 - 1.85]** \| **0.012** \| 1.21 [0.74 - 1.98] \| 0.532 \| 1.17 [0.68 - 2.02] \| 0.656 \| \| **>3 days of fever** \| 29.2% (n = 374) \| 57.1% (n = 151) \| 59.0% (n = 39) \| **3.23 [2.46 - 4.23]** \| **<0.001** \| **3.49 [2.10 - 5.78]** \| **<0.001** \| 0.93 [0.53 - 1.6] \| 0.8893 \| \| **Cough** \| 25.6% (n = 327) \| 33.3% (n = 88) \| 45.4% (n = 30) \| **1.45 [1.09 - 1.93]** \| **0.012** \| **2.42 [1.47 - 3.99]** \| **0.001** \| 0.60 [0.35 - 1.04] \| 0.090 \| \| **Vomiting** \| 33.1% (n = 423) \| 30.6% (n = 81) \| 30.3% (n = 20) \| 0.89 [0.67 - 1.19] \| 0.485 \| 0.88 [0.51 - 1.50] \| 0.733 \| 1.02 [0.57 - 1.83] \| 1 \| \| **Diarrhea** \| 16.0% (n = 205) \| 31.4% (n = 83) \| 33.3% (n = 22) \| **2.40 [1.78 - 3.24]** \| **<0.001** \| **2.61 [1.53 - 4.46]** \| **<0.001** \| 0.92 [0.52 - 1.63] \| 0.883 \| \| **Incapacity to eat/drink** \| 7.1% (n = 91) \| 10.9% (n = 29) \| 9.1% (n = 6) \| **1.61 [1.04 - 2.50]** \| **0.045** \| 1.30 [0.55 - 3.10] \| 0.721 \| 1.23 [0.49 - 3.11] \| 0.823 \| \| **Prehospital antibiotics** \| 28.7% (n = 367) \| 40.5% (n = 107) \| 57.5% (n = 38) \| **1.69 [1.28 - 2.22]** \| **<0.001** \| **3.37 [2.04 - 5.56]** \| **<0.001** \| **0.50 [0.29 - 0.87]** \| **0.018** \| \| **Prehospital antimalarials** \| 32.2% (n = 412) \| 45.8% (n = 121) \| 56.0% (n = 37) \| **1.78 [1.36 - 2.32]** \| **<0.001** \| **2.68 [1.62 - 4.42]** \| **<0.001** \| 0.66 [0.39 - 1.14] \| 0.177 \| \| **Prehospital transfusion** \| 4.4% (n = 56) \| 14.3% (n = 38) \| 12.1% (n = 8) \| **3.67 [2.37 - 5.67]** \| **<0.001** \| **3.01 [1.37 - 6.60]** \| **0.010** \| 1.22 [0.54 - 2.75] \| 0.842 \| \| **Prehospital iron supplements** \| 22.4% (n = 287) \| 28.4% (n = 75) \| 22.7% (n = 15) \| **1.37 [1.02 - 1.84]** \| **0.047** \| 1.01 [0.56 - 1.83] \| 1 \| 1.35 [0.72 - 2.55] \| 0.44 \| \| **Fever on enrollment** \| 42.0% (n = 537) \| 40.5% (n = 107) \| 53.0% (n = 35) \| 0.94 [0.72 - 1.23] \| 0.698 \| 1.56 [0.95 - 2.55] \| 0.103 \| 0.60 [0.35 - 1.04] \| 0.090 \| \| **Tachycardia on enrollment** \| 80.7% (n = 1031) \| 76.1% (n = 201) \| 66.6% (n = 44) \| 0.76 [0.56 - 1.04] \| 0.106 \| **0.48 [0.28 - 0.81]** \| **0.009** \| 1.60 [0.89 - 2.86] \| 0.157 \| \| **Tachypnea on enrollment** \| 75.7% (n = 967) \| 69.3% (n = 183) \| 56.0% (n = 37) \| **0.72 [0.54 - 0.97]** \| **0.036** \| **0.41 [0.25 - 0.68]** \| **0.001** \| 1.77 [1.02 - 3.08] \| 0.058 \| \| **Hypoxia on enrollment** \| 6.2% (n = 79) \| 7.2% (n = 19) \| 18.1% (n = 12) \| 1.18 [0.70 - 1.98] \| 0.635 \| **3.37 [1.73 - 6.56]** \| **0.001** \| **0.35 [0.16 - 0.76]** \| **0.016** \| \| **Moderate acute malnutrition*** \| 10.5% (n = 135) \| 18.5% (n = 49) \| 18.1% (n = 12) \| **2.18 [1.52 - 3.13]** \| **<0.001** \| **2.28 [1.17 - 4.43]** \| **0.024** \| 0.96 [0.47 - 1.96] \| 1 \| \| **Severe acute malnutrition*** \| 5.2% (n = 67) \| 13.6% (n = 36) \| 18.1% (n = 12) \| **3.23 [2.09 - 4.98]** \| **<0.001** \| **4.58 [2.31 - 9.12]** \| **<0.001** \| 0.70 [0.34 - 1.47] \| 0.459 \| \| **Prostration** \| 69.4% (n = 887) \| 65.5% (n = 173) \| 59.0% (n = 39) \| 0.84 [0.63 - 1.11] \| 0.237 \| 0.64 [0.38 - 1.05] \| 0.101 \| 1.32 [0.76 - 2.29] \| 0.405 \| \| **Clinical dehydration** \| 3.0% (n = 38) \| 7.2% (n = 19) \| 9.1% (n = 6) \| **2.53 [1.43 - 4.46]** \| **0.002** \| **3.26 [1.33 - 8.01]** \| **0.018** \| 0.78 [0.30 - 2.03] \| 0.605 \| \| **Jaundice** \| 4.9% (n = 63) \| 12.8% (n = 34) \| 12.1% (n = 8) \| **2.85 [1.83 - 4.42]** \| **<0.001** \| **2.66 [1.22 - 5.81]** \| **0.024** \| 1.07 [0.47 - 2.44] \| 1 \| \| **Not alert** *(AVPU scale: V, P or U)* \| 10.0% (n = 128) \| 14.7% (n = 39) \| 12.1% (n = 8) \| **1.56 [1.06 - 2.29]** \| **0.031** \| 1.24 [0.58 - 2.65] \| 0.733 \| 1.26 [0.56 - 2.84] \| 0.723 \| \| **Convulsions** *(before/on enrollment)* \| 18.0% (n = 230) \| 12.1% (n = 32) \| 15.1% (n = 10) \| **0.63 [0.42 - 0.93]** \| **0.025** \| 0.81 [0.41 - 1.62] \| 0.668 \| 0.77 [0.36 - 1.66] \| 0.650 \| \| **Signs of meningitis** *(bombing fontanel, neck stiffness or irritability)* \| 3.1% (n = 39) \| 2.7% (n = 7) \| 7.6% (n = 5) \| 0.86 [0.38 - 1.95] \| 0.880 \| 2.60 [0.99 - 6.84] \| 0.060 \| 0.33 [0.10 - 1.08] \| 0.069 \| \| **Grunting** \| 18.0% (n = 231) \| 22.7% (n = 60) \| 27.2% (n = 18) \| 1.33 [0.97 - 1.84] \| 0.096 \| 1.70 [0.97 - 2.97] \| 0.087 \| 0.78 [0.42 - 1.45] \| 0.538 \| \| **Labored breathing** *(nasal flaring, chest/intercostal retractions)* \| 25.8% (n = 330) \| 29.9% (n = 79) \| 36.3% (n = 24) \| 1.23 [0.92 - 1.64] \| 0.197 \| 1.64 [0.98 - 2.75] \| 0.080 \| 0.75 [0.42 - 1.32] \| 0.389 \| \| **Hepatomegaly** \| 24.3% (n = 311) \| 40.1% (n = 106) \| 39.3% (n = 26) \| **2.08 [1.58 - 2.75]** \| **<0.001** \| **2.02 [1.21 - 3.36]** \| **0.009** \| 1.03 [0.59 - 1.79] \| 1 \| \| **Splenomegaly** \| 25.2% (n = 323) \| 38.6% (n = 102) \| 36.3% (n = 24) \| **1.86 [1.41 - 2.46]** \| **<0.001** \| 1.69 [1.01 - 2.83] \| 0.063 \| 1.10 [0.63 - 1.93] \| 0.843 \| \| **Moderate anemia** *(Hb <=9.3 g/dl)** \| 75.0% (n = 958) \| 68.5% (n = 181) \| 66.6% (n = 44) \| **0.62 [0.44 - 0.86]** \| **0.006** \| **0.41 [0.24 - 0.71]** \| **0.002** \| 1.49 [0.82 - 2.71] \| 0.250 \| \| **Severe anemia** *(Hb <5 g/dl)** \| 10.1% (n = 130) \| 9.5% (n = 25) \| 1.5% (n = 1) \| 0.63 [0.37 - 1.05] \| 0.100 \| **0.07 [0.01 - 0.52]** \| **<0.001** \| **9.05 [1.15 - 71.03]** \| **0.028** \| \| **Hypoglycemia** *(<45 mg/dl)* \| 2.5% (n = 32) \| 9.1% (n = 24) \| 9.1% (n = 6) \| **3.89 [2.25 - 6.72]** \| **<0.001** \| **3.89 [1.57 - 9.66]** \| **0.009** \| 1.00 [0.39 - 2.56] \| 1 \| \| *Cases with unknown malaria status (n = 1) were excluded from the analysis in this table. * Reference group: no acute malnutrition/no anemia* \| \| \| \| \| \| \| \| \| \|   **Table F.** Comparative clinical presentation of children with non-typhoidal *Salmonella* (NTS) bloodstream infection (BSI) according to current, recent, or no *Plasmodium falciparum* (*Pf*) malaria co-infection. | | | | | | | | | |
| --- | --- | --- | --- | --- | --- | --- | --- | --- | --- | --- | --- | --- | --- | --- | --- | --- | --- | --- | --- | --- | --- | --- | --- | --- | --- | --- | --- | --- | --- | --- | --- | --- | --- | --- | --- | --- | --- | --- | --- | --- | --- | --- | --- | --- | --- | --- | --- | --- | --- | --- | --- | --- | --- | --- | --- | --- | --- | --- | --- | --- | --- | --- | --- | --- | --- | --- | --- | --- | --- | --- | --- | --- | --- | --- | --- | --- | --- | --- | --- | --- | --- | --- | --- | --- | --- | --- | --- | --- | --- | --- | --- | --- | --- | --- | --- | --- | --- | --- | --- | --- | --- | --- | --- | --- | --- | --- | --- | --- | --- | --- | --- | --- | --- | --- | --- | --- | --- | --- | --- | --- | --- | --- | --- | --- | --- | --- | --- | --- | --- | --- | --- | --- | --- | --- | --- | --- | --- | --- | --- | --- | --- | --- | --- | --- | --- | --- | --- | --- | --- | --- | --- | --- | --- | --- | --- | --- | --- | --- | --- | --- | --- | --- | --- | --- | --- | --- | --- | --- | --- | --- | --- | --- | --- | --- | --- | --- | --- | --- | --- | --- | --- | --- | --- | --- | --- | --- | --- | --- | --- | --- | --- | --- | --- | --- | --- | --- | --- | --- | --- | --- | --- | --- | --- | --- | --- | --- | --- | --- | --- | --- | --- | --- | --- | --- | --- | --- | --- | --- | --- | --- | --- | --- | --- | --- | --- | --- | --- | --- | --- | --- | --- | --- | --- | --- | --- | --- | --- | --- | --- | --- | --- | --- | --- | --- | --- | --- | --- | --- | --- | --- | --- | --- | --- | --- | --- | --- | --- | --- | --- | --- | --- | --- | --- | --- | --- | --- | --- | --- | --- | --- | --- | --- | --- | --- | --- | --- | --- | --- | --- | --- | --- | --- | --- | --- | --- | --- | --- | --- | --- | --- | --- | --- | --- | --- | --- | --- | --- | --- | --- | --- | --- | --- | --- | --- | --- | --- | --- | --- | --- | --- | --- | --- | --- | --- | --- | --- | --- | --- | --- | --- | --- | --- | --- | --- | --- | --- | --- | --- | --- | --- | --- | --- | --- | --- | --- | --- | --- | --- | --- | --- | --- | --- | --- | --- | --- | --- | --- | --- | --- | --- | --- | --- | --- | --- | --- | --- | --- | --- | --- | --- | --- | --- | --- | --- | --- |
|  | **% in NTS BSI**  **+ current *Pf*** (n = 91) | **% in NTS BSI**  **+ recent *Pf*** (n = 173) | **% in NTS BSI**  **without *Pf*** (n = 66) | **NTS BSI with recent *Pf***  **vs NTS BSI with current *Pf*** | | **NTS BSI without *Pf***  **vs NTS BSI with current *Pf*** | | **NTS BSI without *Pf***  **vs NTS BSI with recent *Pf*** | |
|  |  |  |  | OR [95% CI] | p-value | OR [95% CI] | p-value | OR [95% CI] | p-value |
| **Female sex** | 58.2% (n = 53) | 47.3% (n = 82) | 34.8% (n = 23) | 0.65 [0.39 - 1.08] | 0.122 | **0.38 [0.20 - 0.74]** | **0.006** | 0.59 [0.33 - 1.07] | 0.109 |
| **Age under 2 years** | 75.8% (n = 69) | 69.3% (n = 120) | 93.9% (n = 62) | 0.72 [0.40 - 1.29] | 0.336 | **4.94 [1.61 - 15.1]** | **0.005** | **6.85 [2.37 - 19.8]** | **<0.001** |
| **Residence in rural village** | 9.89% (n = 9) | 16.1% (n = 28) | 16.6% (n = 11) | 1.76 [0.79 - 3.91] | 0.225 | 1.82 [0.71 - 4.69] | 0.31 | 1.04 [0.48 - 2.22] | 1 |
| **Previous hospital admission** *(in 12 months before fever onset)* | 56.6% (n = 51) | 54.9% (n = 95) | 51.5% (n = 34) | 0.93 [0.56 - 1.56] | 0.888 | 0.81 [0.43 - 1.54] | 0.634 | 0.87 [0.49 - 1.54] | 0.744 |
| **>3 days of fever** | 41.7% (n = 38) | 65.3% (n = 113) | 59.0% (n = 39) | **2.63 [1.56 - 4.42]** | **<0.001** | **2.01 [1.06 - 3.83]** | **0.047** | 0.77 [0.43 - 1.37] | 0.4568 |
| **Cough** | 29.6% (n = 27) | 35.2% (n = 61) | 45.4% (n = 30) | 1.29 [0.75 - 2.23] | 0.436 | 1.98 [1.02 - 3.83] | 0.063 | 1.53 [0.86 - 2.72] | 0.193 |
| **Vomiting** | 34.0% (n = 31) | 28.9% (n = 50) | 30.3% (n = 20) | 0.79 [0.46 - 1.36] | 0.469 | 0.84 [0.43 - 1.66] | 0.746 | 1.07 [0.58 - 1.99] | 0.957 |
| **Diarrhea** | 19.7% (n = 18) | 37.5% (n = 65) | 33.3% (n = 22) | **2.44 [1.34 - 4.45]** | **0.005** | 2.03 [0.98 - 4.19] | 0.082 | 0.83 [0.46 - 1.51] | 0.647 |
| **Incapacity to eat/drink** | 8.79% (n = 8) | 12.1% (n = 21) | 9.09% (n = 6) | 1.43 [0.61 - 3.38] | 0.536 | 1.04 [0.34 - 3.15] | 1 | 0.72 [0.28 - 1.88] | 0.662 |
| **Prehospital antibiotics** | 27.4% (n = 25) | 47.3% (n = 82) | 57.5% (n = 38) | **2.38 [1.37 - 4.12]** | **0.003** | **3.58 [1.83 - 7.01]** | **<0.001** | 1.51 [0.85 - 2.67] | 0.207 |
| **Prehospital antimalarials** | 28.5% (n = 26) | 54.9% (n = 95) | 56.0% (n = 37) | **3.04 [1.77 - 5.25]** | **<0.001** | **3.19 [1.64 - 6.21]** | **0.001** | 1.05 [0.59 - 1.85] | 0.989 |
| **Prehospital transfusion** | 3.29% (n = 3) | 20.2% (n = 35) | 12.1% (n = 8) | **7.44 [2.22 - 24.92]** | **<0.001** | 4.05 [1.03 - 15.9] | 0.054 | 0.54 [0.24 - 1.24] | 0.204 |
| **Prehospital iron supplements** | 25.2% (n = 23) | 30.0% (n = 52) | 22.7% (n = 15) | 1.27 [0.72 - 2.26] | 0.499 | 0.87 [0.41 - 1.83] | 0.858 | 0.68 [0.35 - 1.33] | 0.334 |
| **Fever on enrollment** | 48.3% (n = 44) | 36.4% (n = 63) | 53.0% (n = 35) | 0.61 [0.37 - 1.02] | 0.081 | 1.21 [0.64 - 2.27] | 0.677 | **1.97 [1.11 - 3.50]** | **0.029** |
| **Tachycardia on enrollment** | 83.5% (n = 76) | 72.2% (n = 125) | 66.6% (n = 44) | 0.51 [0.27 - 0.98] | 0.059 | **0.39 [0.19 - 0.84]** | **0.024** | 0.77 [0.42 - 1.41] | 0.49 |
| **Tachypnea on enrollment** | 79.1% (n = 72) | 64.1% (n = 111) | 56.0% (n = 37) | **0.47 [0.26 - 0.86]** | **0.018** | **0.34 [0.17 - 0.68]** | **0.003** | 0.71 [0.40 - 1.27] | 0.315 |
| **Hypoxia on enrollment** | 6.59% (n = 6) | 7.51% (n = 13) | 18.1% (n = 12) | 1.15 [0.42 - 3.14] | 0.98 | **3.15 [1.12 - 8.89]** | **0.04** | **2.74 [1.18 - 6.35]** | **0.03** |
| **Moderate acute malnutrition** | 16.4% (n = 15) | 19.6% (n = 34) | 18.1% (n = 12) | 1.23 [0.62 - 2.43] | 0.668* | 1.20 [0.51 - 2.82] | 0.842* | 0.97 [0.46 - 2.06] | 1* |
| **Severe acute malnutrition** | 14.2% (n = 13) | 13.2% (n = 23) | 18.1% (n = 12) | 0.96 [0.46 - 2.03] | 1* | 1.38 [0.58 - 3.33] | 0.504* | 1.44 [0.66 - 3.15] | 0.477* |
| **Prostration** | 57.1% (n = 52) | 69.9% (n = 121) | 59.0% (n = 39) | 1.75 [1.03 - 2.96] | 0.052 | 1.08 [0.57 - 2.06] | 0.936 | 0.62 [0.34 - 1.12] | 0.15 |
| **Clinical dehydration** | 5.49% (n = 5) | 8.09% (n = 14) | 9.09% (n = 6) | 1.51 [0.53 - 4.35] | 0.599 | 1.72 [0.50 - 5.89] | 0.529 | 1.14 [0.42 - 3.09] | 1 |
| **Jaundice** | 8.79% (n = 8) | 15.0% (n = 26) | 12.1% (n = 8) | 1.84 [0.79 - 4.24] | 0.213 | 1.43 [0.51 - 4.03] | 0.679 | 0.78 [0.33 - 1.82] | 0.713 |
| **Not alert** *(AVPU scale: V, P or U)* | 8.79% (n = 8) | 17.9% (n = 31) | 12.1% (n = 8) | 2.26 [0.99 - 5.16] | 0.071 | 1.43 [0.51 - 4.03] | 0.679 | 0.63 [0.27 - 1.46] | 0.374 |
| **Convulsions** *(before/on enrollment)* | 12.0% (n = 11) | 12.1% (n = 21) | 15.1% (n = 10) | 1.00 [0.46 - 2.19] | 1 | 1.30 [0.52 - 3.27] | 0.75 | 1.29 [0.57 - 2.91] | 0.686 |
| **Signs of meningitis** *(bombing fontanel, neck stiffness or irritability)* | 0% (n = 0) | 4.04% (n = 7) | 7.57% (n = 5) | NA | 0.1 | NA | **0.012** | 1.94 [0.59 - 6.35] | 0.32 |
| **Grunting** | 19.7% (n = 18) | 24.2% (n = 42) | 27.2% (n = 18) | 1.30 [0.70 - 2.42] | 0.5 | 1.52 [0.72 - 3.21] | 0.363 | 1.17 [0.61 - 2.23] | 0.756 |
| **Labored breathing** *(nasal flaring, chest/intercostal retractions)* | 25.2% (n = 23) | 32.3% (n = 56) | 36.3% (n = 24) | 1.42 [0.80 - 2.5] | 0.291 | 1.69 [0.85 - 3.37] | 0.186 | 1.19 [0.66 - 2.16] | 0.666 |
| **Hepatomegaly** | 37.3% (n = 34) | 41.6% (n = 72) | 39.3% (n = 26) | 1.20 [0.71 - 2.01] | 0.59 | 1.09 [0.57 - 2.09] | 0.927 | 0.91 [0.51 - 1.63] | 0.869 |
| **Splenomegaly** | 35.1% (n = 32) | 40.4% (n = 70) | 36.3% (n = 24) | 1.25 [0.74 - 2.12] | 0.479 | 1.05 [0.54 - 2.04] | 1 | 0.84 [0.47 - 1.51] | 0.666 |
| **Moderate anemia** *(Hb <=9.3 g/dl)* | 73.6% (n = 67) | 65.8% (n = 114) | 66.6% (n = 44) | 0.49 [0.25 - 0.98] | 0.059* | **0.41 [0.18 - 0.90]** | **0.038*** | 0.83 [0.44 - 1.54] | 0.663* |
| **Severe anemia** *(Hb <5 g/dl)* | 12.0% (n = 11) | 8.09% (n = 14) | 1.51% (n = 1) | 0.37 [0.13 – 1.00] | 0.084* | **0.06 [0.01 - 0.49]** | **0.004*** | 0.15 [0.02 - 1.24] | 0.057* |
| **Hypoglycemia** *(<45 mg/dl)* | 6.59% (n = 6) | 10.4% (n = 18) | 9.09% (n = 6) | 1.65 [0.63 - 4.30] | 0.425 | 1.42 [0.44 - 4.61] | 0.561 | 0.86 [0.33 - 2.27] | 1 |
| *Cases with unknown malaria status (n = 1) were excluded from the analysis in this table. * Reference group: no acute malnutrition/no anemia* | | | | | | | | | |

| **Table G.** Comparison of clinical presentation of children with non-typhoidal *Salmonella* (NTS) bloodstream infection (BSI) according to serotype. | | | | | | | | | |
| --- | --- | --- | --- | --- | --- | --- | --- | --- | --- |
|  | **% in O5-positive Typhimurium** (n = 163) | **% in O5-negative Typhimurium** (n = 108) | **% in Enteritidis**  (n = 54) | **O5-positive Typhimurium  vs Enteritidis** | | **O5-negative Typhimurium vs Enteritidis** | | **O5-negative vs O5-positive Typhimurium** | |
|  |  |  |  | OR [95% CI] | p-value | OR [95% CI] | p-value | OR [95% CI] | p-value |
| **Female sex** | 52.7% (n = 86) | 45.3% (n = 49) | 40.7% (n = 22) | 1.62 [0.87 - 3.03] | 0.169 | 1.21 [0.62 - 2.34] | 0.695 | 0.74 [0.46 - 1.21] | 0.286 |
| **Age under 2 years** | 77.3% (n = 126) | 77.7% (n = 84) | 70.3% (n = 38) | 1.43 [0.72 - 2.86] | 0.398 | 1.47 [0.70 - 3.09] | 0.402 | 1.03 [0.57 - 1.84] | 1 |
| **Residence in rural village** | 19.0% (n = 31) | 9.25% (n = 10) | 12.9% (n = 7) | 1.58 [0.65 - 3.82] | 0.419 | 0.69 [0.25 - 1.91] | 0.650 | **0.43 [0.20 - 0.93]** | **0.043** |
| **Previous hospital admission** *(in 12 months before fever onset)* | 56.4% (n = 92) | 55.5% (n = 60) | 45.2% (n = 24) | 1.57 [0.84 - 2.92] | 0.209 | 1.51 [0.78 - 2.92] | 0.290 | 0.96 [0.59 - 1.57] | 0.985 |
| **>3 days of fever** | 59.5% (n = 97) | 55.5% (n = 60) | 57.4% (n = 31) | 1.09 [0.58 - 2.03] | 0.9104 | 0.93 [0.48 - 1.79] | 0.956 | 0.85 [0.52 - 1.39] | 0.6032 |
| **Cough** | 36.8% (n = 60) | 37.9% (n = 41) | 25.9% (n = 14) | 1.66 [0.84 - 3.31] | 0.195 | 1.75 [0.85 - 3.60] | 0.177 | 1.05 [0.64 - 1.74] | 0.949 |
| **Vomiting** | 30.0% (n = 49) | 35.1% (n = 38) | 24.0% (n = 13) | 1.36 [0.67 - 2.75] | 0.503 | 1.71 [0.82 - 3.58] | 0.209 | 1.26 [0.75 - 2.12] | 0.452 |
| **Diarrhea** | 31.2% (n = 51) | 33.3% (n = 36) | 27.7% (n = 15) | 1.18 [0.60 - 2.34] | 0.752 | 1.30 [0.63 - 2.66] | 0.590 | 1.10 [0.65 - 1.85] | 0.826 |
| **Incapacity to eat/drink** | 9.81% (n = 16) | 8.33% (n = 9) | 18.5% (n = 10) | 0.48 [0.20 - 1.13] | 0.143 | 0.40 [0.15 - 1.05] | 0.101 | 0.84 [0.36 - 1.96] | 0.843 |
| **Prehospital antibiotics** | 45.3% (n = 74) | 41.6% (n = 45) | 42.5% (n = 23) | 1.12 [0.60 - 2.09] | 0.84 | 0.96 [0.50 - 1.87] | 1 | 0.86 [0.53 - 1.40] | 0.630 |
| **Prehospital antimalarials** | 48.4% (n = 79) | 42.5% (n = 46) | 53.7% (n = 29) | 0.81 [0.44 - 1.50] | 0.61 | 0.64 [0.33 - 1.23] | 0.242 | 0.79 [0.48 - 1.29] | 0.409 |
| **Prehospital transfusion** | 21.4% (n = 35) | 7.40% (n = 8) | 3.70% (n = 2) | **7.11 [1.65 - 30.6]** | **0.005** | 2.08 [0.43 - 10.2] | 0.498 | **0.29 [0.13 - 0.66]** | **0.003** |
| **Prehospital iron supplements** | 31.2% (n = 51) | 22.2% (n = 24) | 25.9% (n = 14) | 1.30 [0.65 - 2.60] | 0.566 | 0.82 [0.38 - 1.74] | 0.743 | 0.63 [0.36 - 1.10] | 0.135 |
| **Fever on enrollment** | 42.3% (n = 69) | 46.2% (n = 50) | 38.8% (n = 21) | 1.15 [0.61 - 2.16] | 0.775 | 1.35 [0.70 - 2.63] | 0.467 | 1.17 [0.72 - 1.92] | 0.604 |
| **Tachycardia on enrollment** | 70.5% (n = 115) | 79.6% (n = 86) | 74.0% (n = 40) | 0.84 [0.42 - 1.68] | 0.747 | 1.37 [0.63 - 2.95] | 0.548 | 1.63 [0.92 - 2.90] | 0.126 |
| **Tachypnea on enrollment** | 65.0% (n = 106) | 71.2% (n = 77) | 59.2% (n = 32) | 1.28 [0.68 - 2.40] | 0.548 | 1.71 [0.86 - 3.39] | 0.173 | 1.34 [0.79 - 2.26] | 0.344 |
| **Hypoxia on enrollment** | 10.4% (n = 17) | 10.1% (n = 11) | 5.55% (n = 3) | 1.98 [0.56 - 7.04] | 0.417 | 1.93 [0.51 - 7.22] | 0.389 | 0.97 [0.44 - 2.17] | 1 |
| **Moderate acute malnutrition** | 20.2% (n = 33) | 18.5% (n = 20) | 12.9% (n = 7) | 1.62 [0.66 - 3.96] | 0.398* | 1.47 [0.57 - 3.79] | 0.570* | 0.91 [0.48 - 1.71] | 0.891* |
| **Severe acute malnutrition** | 13.4% (n = 22) | 14.8% (n = 16) | 18.5% (n = 10) | 0.75 [0.33 - 1.74] | 0.657* | 0.82 [0.34 - 1.99] | 0.837* | 1.09 [0.54 - 2.22] | 0.953* |
| **Prostration** | 61.9% (n = 101) | 65.7% (n = 71) | 66.6% (n = 36) | 0.81 [0.43 - 1.56] | 0.647 | 0.96 [0.48 - 1.92] | 1 | 1.18 [0.71 - 1.96] | 0.615 |
| **Clinical dehydration** | 8.58% (n = 14) | 5.55% (n = 6) | 5.55% (n = 3) | 1.60 [0.44 - 5.78] | 0.572 | 1.00 [0.24 - 4.16] | 1 | 0.63 [0.23 - 1.68] | 0.485 |
| **Jaundice** | 14.7% (n = 24) | 12.9% (n = 14) | 7.40% (n = 4) | 2.16 [0.71 - 6.53] | 0.248 | 1.86 [0.58 - 5.96] | 0.426 | 0.86 [0.42 - 1.75] | 0.818 |
| **Not alert** *(AVPU scale: V, P or U)* | 16.5% (n = 27) | 12.9% (n = 14) | 11.1% (n = 6) | 1.59 [0.62 - 4.08] | 0.454 | 1.19 [0.43 - 3.30] | 0.933 | 0.75 [0.37 - 1.51] | 0.524 |
| **Convulsions** *(before/on enrollment)* | 14.1% (n = 23) | 9.25% (n = 10) | 12.9% (n = 7) | 1.10 [0.44 - 2.74] | 1 | 0.69 [0.25 - 1.91] | 0.650 | 0.62 [0.28 - 1.36] | 0.314 |
| **Signs of meningitis** *(bombing fontanel, neck stiffness or irritability)* | 5.52% (n = 9) | 0.92% (n = 1) | 1.85% (n = 1) | 3.1 [0.38 - 25.03] | 0.457 | 0.50 [0.03 - 8.07] | 1 | 0.16 [0.02 - 1.28] | 0.055 |
| **Grunting** | 20.8% (n = 34) | 23.1% (n = 25) | 31.4% (n = 17) | 0.57 [0.29 - 1.14] | 0.158 | 0.66 [0.32 - 1.36] | 0.342 | 1.14 [0.64 - 2.05] | 0.767 |
| **Labored breathing** *(nasal flaring, chest/intercostal retractions)* | 27.6% (n = 45) | 42.5% (n = 46) | 22.2% (n = 12) | 1.33 [0.64 - 2.76] | 0.548 | **2.60 [1.23 - 5.48]** | **0.018** | **1.95 [1.16 - 3.25]** | **0.015** |
| **Hepatomegaly** | 39.8% (n = 65) | 41.6% (n = 45) | 35.1% (n = 19) | 1.22 [0.64 - 2.32] | 0.651 | 1.32 [0.67 - 2.59] | 0.532 | 1.08 [0.66 - 1.77] | 0.867 |
| **Splenomegaly** | 36.8% (n = 60) | 40.7% (n = 44) | 35.1% (n = 19) | 1.07 [0.56 - 2.04] | 0.959 | 1.27 [0.64 - 2.49] | 0.608 | 1.18 [0.72 - 1.94] | 0.600 |
| **Moderate anemia** *(Hb <=9.3 g/dl)* | 64.4% (n = 105) | 72.2% (n = 78) | 72.2% (n = 39) | 0.56 [0.26 - 1.22] | 0.195* | 1.05 [0.45 - 2.48] | 1* | 1.88 [1.02 - 3.44] | 0.057* |
| **Severe anemia** *(Hb <5 g/dl)* | 6.13% (n = 10) | 10.1% (n = 11) | 9.25% (n = 5) | 0.42 [0.12 - 1.49] | 0.279* | 1.16 [0.31 - 4.27] | 1* | 2.78 [1.01 - 7.61] | 0.078* |
| **Hypoglycemia** *(<45 mg/dl)* | 7.97% (n = 13) | 9.25% (n = 10) | 12.9% (n = 7) | 0.58 [0.22 - 1.54] | 0.283 | 0.69 [0.25 - 1.91] | 0.587 | 1.18 [0.50 - 2.79] | 0.882 |
| ** Reference group: no acute malnutrition/no anemia* | | | | | | | | | |

| **Table H.** Case fatality and timing of death according to presence of culture-confirmed bloodstream infection (BSI) and malaria status. | | |
| --- | --- | --- |
|  | **Case fatality ratio** | **Proportion of deaths by day 2** |
| **Severe *Pf* malaria without BSI** | 2.9% (37/1277) | 89.2% (33/37) |
| **NTS - *Pf* malaria co-infection** | 22.0% (58/264) | 65.5% (38/58) |
| *NTS with current Pf malaria* | 18.7% (17/91) | 52.9% (9/17) |
| *NTS with recent Pf malaria* | 23.7% (41/173) | 70.7% (29/41) |
| **NTS BSI without malaria** | 31.3% (21/66) | 61.9% (13/21) |
| **Other BSI** | 26.4% (19/72) | 73.7% (14/19) |
| *Other BSI without Pf malaria* | 33.3% (7/21) | 57.1% (4/7) |
| *Other BSI + current Pf malaria* | 20.6% (7/34) | 85.7% (6/7) |
| *Other BSI + recent Pf malaria* | 29.4% (5/17) | 80.0% (4/5) |
| **Other febrile illness** | 4.8% (48/999) | 54.2% (26/48) |
| *Uncomplicated Pf malaria without BSI* | 0.7% (2/298) | 50.0% (1/2) |
| *Recent Pf malaria without BSI* | 5.9% (21/355) | 52.4% (11/21) |
| *Current non-Pf malaria without BSI* | 7.7% (1/13) | 100.0% (1/1) |
| *No malaria + no BSI* | 7.2% (24/333) | 54.2% (13/24) |
| **Overall** | **6.9% (185/2682)** | **67.6% (125/185)** |
| *Abbreviations: NTS: non-typhoidal Salmonella, Pf: Plasmodium falciparum* | | |

| **Fig B.** Overall (in-hospital and post-discharge) survival analysis according to diagnostic strata. |
| --- |
| 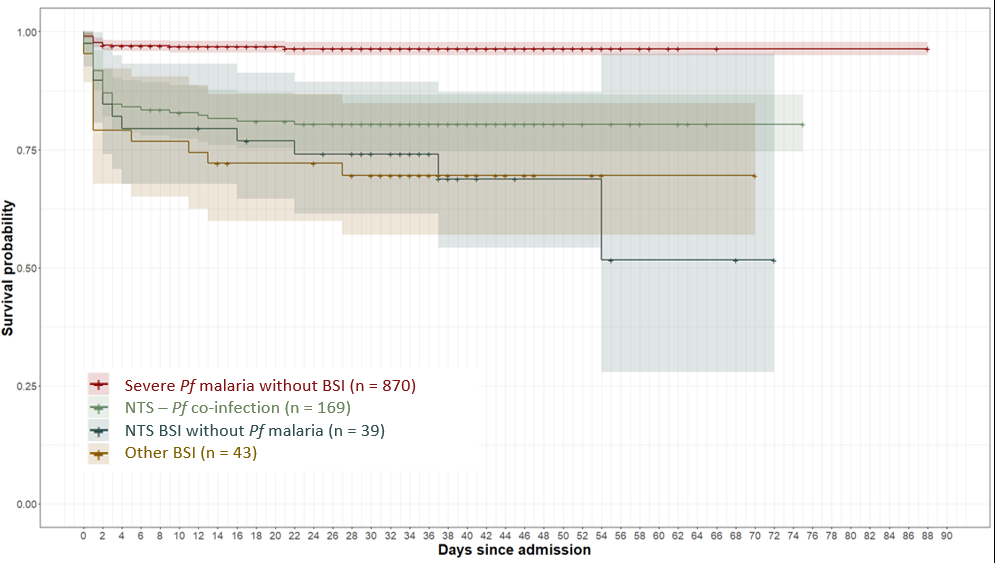 |
| *Data were available for the DeNTS period (February 2021 – January 2022). Abbreviations: NTS: non-typhoidal Salmonella, BSI: bloodstream infection, Pf: Plasmodium falciparum* |

| **Fig C.** Seasonal distribution of cases and deaths per diagnostic category. |
| --- |
| 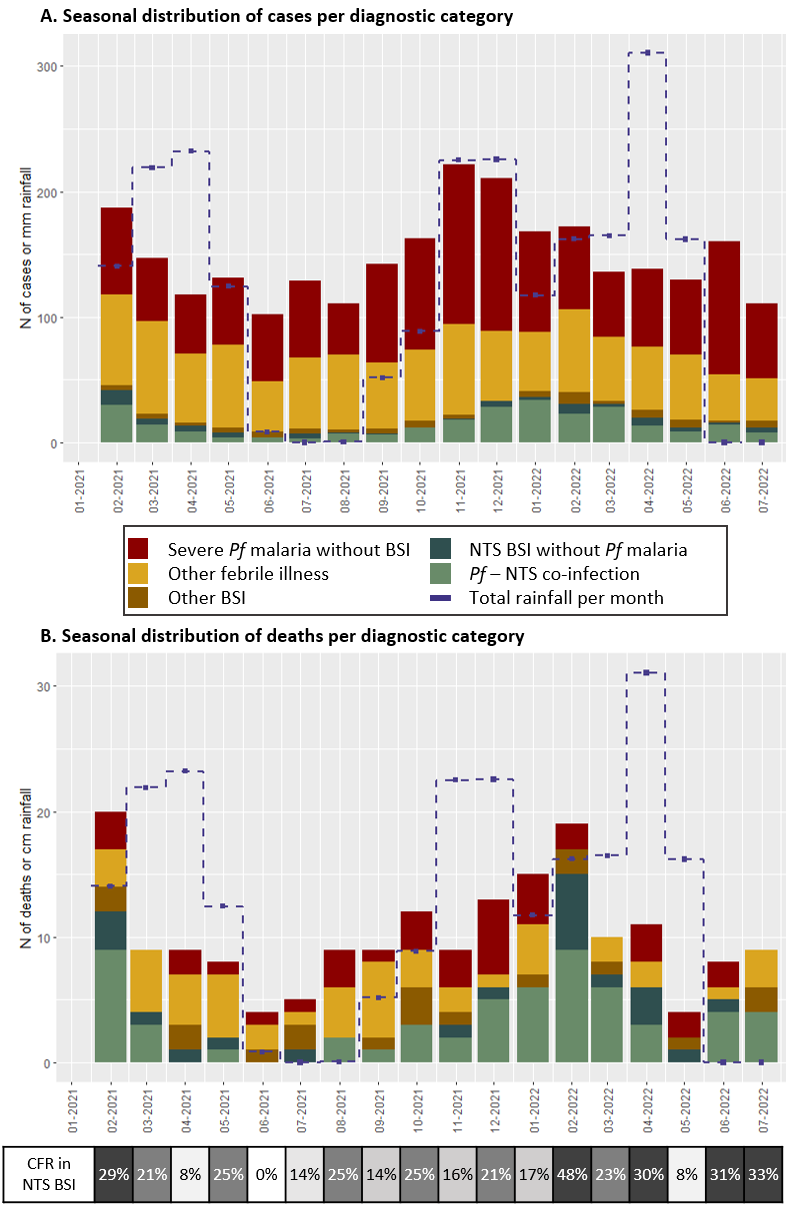 |
| *Bars indicate the number of cases/deaths and are colored according to diagnostic category. The blue stepwise graph indicates the total rainfall in mm per month, remotely estimated (CHIRPS daily) and averaged for Kisantu health district. Case fatality ratios (CFR) for non-typhoidal Salmonella (NTS) bloodstream infection (BSI) are demonstrated at the bottom with higher CFR in darker shades.* |

| **Fig D.** Seasonal distribution of NTS cases according to serotype. |
| --- |
| 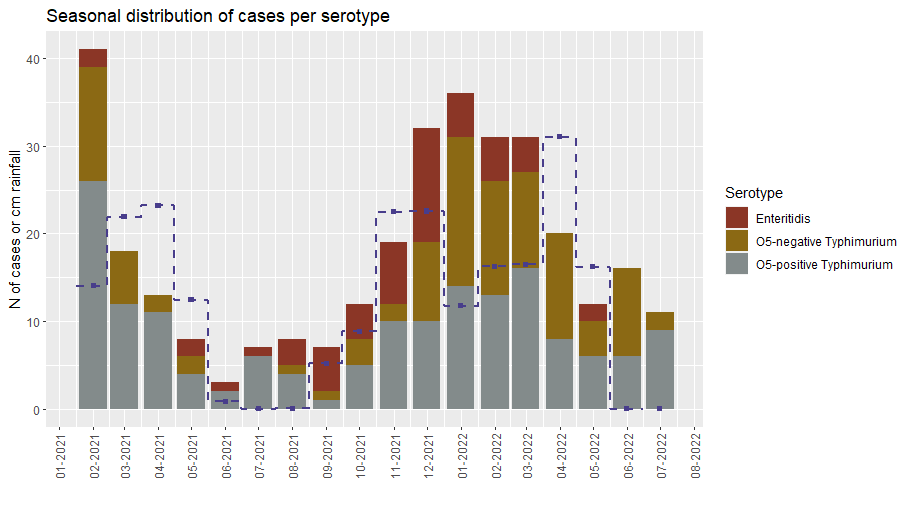 |
| *Bars indicate the number of cases and are colored according to NTS serotype. The blue stepwise graph indicates the total rainfall in mm per month, remotely estimated (CHIRPS daily) and averaged for Kisantu health district.* |

**Table I.** Systematic literature review of studies presenting data on clinical presentation of children with non-typhoidal *Salmonella* (NTS) bloodstream infection in sub-Saharan Africa [10–32]. The PubMed search string was: *Salmonella* [TIAB] AND (non-typh*[TIAB] OR nontyph*[TIAB] OR typhimurium[TIAB] OR enteritidis[TIAB]) AND (bacterem*[TIAB] OR bacteraem*[TIAB] OR "blood culture"[TIAB] OR "bloodstream infection"[TIAB] OR sepsis [TIAB] OR invasive [TIAB])). Systematic review of studies presenting treatment efficacy data for NTS bloodstream infection was previously published by Tack et al. in BMC Medicine in 2020 [33].

This table is available online via <https://figshare.com/s/c83a249c1b36efc55167>

**References:**

1. Tack B, Phoba M-F, Barbé B, Kalonji LM, Hardy L, Van Puyvelde S, et al. Non-typhoidal Salmonella bloodstream infections in Kisantu, DR Congo: Emergence of O5-negative Salmonella Typhimurium and extensive drug resistance. Marks F, editor. PLoS Negl Trop Dis. 2020;14: e0008121. doi:10.1371/journal.pntd.0008121

2. Tack B, Phoba M-F, Van Puyvelde S, Kalonji LM, Hardy L, Barbé B, et al. Salmonella Typhi From Blood Cultures in the Democratic Republic of the Congo: A 10-Year Surveillance. Clinical Infectious Diseases. 2019;68: S130–S137. doi:10.1093/cid/ciy1116

3. Kalonji LM, Post A, Phoba M-F, Falay D, Ngbonda D, Muyembe J-J, et al. Invasive Salmonella Infections at Multiple Surveillance Sites in the Democratic Republic of the Congo, 2011–2014. Clinical Infectious Diseases. 2015;61: S346–S353. doi:10.1093/cid/civ713

4. Lunguya O, Lejon V, Phoba M-F, Bertrand S, Vanhoof R, Glupczynski Y, et al. Antimicrobial resistance in invasive non-typhoid Salmonella from the Democratic Republic of the Congo: emergence of decreased fluoroquinolone susceptibility and extended-spectrum beta lactamases. PLoS Negl Trop Dis. 2013;7: e2103. doi:10.1371/journal.pntd.0002103

5. Clinical and Laboratory Standards Institute. Performance Standards for Antimicrobial Susceptibility Testing supplement M100. 31th edition. Clinical and Laboratory Standards Institute, editor. Wayne: Clinical and Laboratory Standards Institute; 2023.

6. World Health Organization. Guidelines for malaria. Geneva; 2023 Mar. Available: https://app.magicapp.org/#/guideline/7089

7. The European Committee on Antimicrobial Susceptibility Testing. The European Committee on Antimicrobial Susceptibility Testing. Basel; 2023 Jan. Available: https://www.eucast.org/expert_rules_and_expected_phenotypes/expected_phenotypes

8. Auzin A, Spits M, Tacconelli E, Rodríguez-Baño J, Hulscher M, Adang E, et al. What is the evidence base of used aggregated antibiotic resistance percentages to change empirical antibiotic treatment? A scoping review. Clinical Microbiology and Infection. 2022;28: 928–935. doi:10.1016/J.CMI.2021.12.003

9. Chang CM, Hsieh MS, Yang CJ, How CK, Chen PC, Meng YH. Effects of empiric antibiotic treatment based on hospital cumulative antibiograms in patients with bacteraemic sepsis: a retrospective cohort study. Clinical Microbiology and Infection. 2023;29: 765–771. doi:10.1016/j.cmi.2023.01.004

10. Muthumbi E, Morpeth SC, Ooko M, Mwanzu A, Mwarumba S, Mturi N, et al. Invasive Salmonellosis in Kilifi, Kenya. Clinical Infectious Diseases. 2015;61: S290–S301. doi:10.1093/cid/civ737

11. Mandomando I, MacEte E, Sigaúque B, Morais L, Quintó L, Sacarlal J, et al. Invasive non-typhoidal Salmonella in Mozambican children. Tropical Medicine and International Health. 2009;14: 1467–1474. doi:10.1111/j.1365-3156.2009.02399.x

12. Enwere G, Biney E, Cheung Y, Zaman SMA, Okoko B, Oluwalana C, et al. Epidemiologic and Clinical Characteristics of Community-Acquired Invasive Bacterial Infections in Children Aged 2???29 Months in The Gambia. Pediatr Infect Dis J. 2006;25: 700–705. doi:10.1097/01.inf.0000226839.30925.a5

13. Brent A, Ahmed I, Ndiritu M, Lewa P, Ngetsa C, Lowe B, et al. Incidence of clinically significant bacteraemia in children who present to hospital in Kenya: community-based observational study. The Lancet. 2006;367: 482–488. doi:10.1016/S0140-6736(06)68180-4

14. Msemo OA, Mbwana J, Mahende C, Malabeja A, Gesase S, Crump JA, et al. Epidemiology and antimicrobial susceptibility of salmonella enterica bloodstream isolates among febrile children in a rural district in Northeastern Tanzania: A cross-sectional study. Clinical Infectious Diseases. 2019;68: S177–S182. doi:10.1093/cid/ciy1126

15. Mtove G, Nadjm B, Amos B, Hendriksen ICE, Muro F, Reyburn H. Use of an HRP2-based rapid diagnostic test to guide treatment of children admitted to hospital in a malaria-endemic area of north-east Tanzania. Tropical Medicine and International Health. 2011;16: 545–550. doi:10.1111/J.1365-3156.2011.02737.X

16. Graham SM, Mwenechanya J, Tembo M, Kabudula M, Molyneux EM, Walsh AL, et al. The pattern of bacteraemia in children with severe malaria. Malawi Medical Journal. 2002;14: 11–15.

17. Mandomando I, Bassat Q, Sigaúque B, Massora S, Quintó L, Ácacio S, et al. Invasive Salmonella Infections Among Children From Rural Mozambique, 2001-2014. Clinical Infectious Diseases. 2015;61: S339–S345. doi:10.1093/cid/civ712

18. Falay D, Maria L, Kuijpers F, Phoba M, Boeck H De, Lunguya O, et al. Microbiological, clinical and molecular findings of non-typhoidal Salmonella bloodstream infections associated with malaria, Oriental Province, Democratic Republic of the Congo. BMC Infect Dis. 2016;16: 1–14. doi:10.1186/s12879-016-1604-1

19. Nesbitt A, Mirza NB. Salmonella Septicaemias in Kenyan Children. J Trop Pediatr. 1989;35. doi:10.1093/tropej/35.1.35

20. Mabey DCW, Brown A, Greenwood BM. Plasmodium falciparum malaria and salmonella infections in gambian children. Journal of Infectious Diseases. 1987;155: 1319–1321. doi:10.1093/infdis/155.6.1319

21. Green SDR, Cheesbrough JS. Salmonella bacteraemia among young children at a rural hospital in western Zaire. Ann Trop Paediatr. 1993;13: 45–53. doi:10.1080/02724936.1993.11747624

22. Lepage P, Bogaerts J, Van Goethem C, Hitimana DG, Nsengumuremyi F. Multiresistant Salmonella typhimurium systemic infection in Rwanda. Clinical features and treatment with cefotaxime. Journal of Antimicrobial Chemotherapy. 1990;26 Suppl A: 53–7. doi:10.1093/jac/26.suppl_a.53

23. Walsh AL, Phiri AJ, Graham SM, Molyneux EM, Molyneux ME. Bacteremia in febrile Malawian children: Clinical and microbiologic features. Pediatric Infectious Disease Journal. 2000;19: 312–318. doi:10.1097/00006454-200004000-00010

24. Graham SM, Walsh AL, Molyneux EM, Phiri AJ, Molyneux ME. Clinical presentation of non-typhoidal Salmonella bacteraemia in Malawian children. Trans R Soc Trop Med Hyg. 2000;94: 310–314. doi:10.1016/S0035-9203(00)90337-7

25. Kariuki S, Revathi G, Kariuki N, Kiiru J, Mwituria J, Hart CA. Characterisation of community acquired non-typhoidal Salmonella from bacteraemia and diarrhoeal infections in children admitted to hospital in Nairobi, Kenya. BMC Microbiol. 2006;6. doi:10.1186/1471-2180-6-101

26. Bassat Q, Guinovart C, Sigaúque B, Mandomando I, Aide P, Sacarlal J, et al. Severe malaria and concomitant bacteraemia in children admitted to a rural Mozambican hospital. Tropical Medicine & International Health. 2009;14: 1011–1019. doi:10.1111/J.1365-3156.2009.02326.X

27. MacLennan CA, Msefula CL, Gondwe EN, Gilchrist JJ, Pensulo P, Mandala WL, et al. Presentation of life-threatening invasive nontyphoidal Salmonella disease in Malawian children: A prospective observational study. PLoS Negl Trop Dis. 2017;11: e0006027. doi:10.1371/journal.pntd.0006027

28. Biggs HM, Lester R, Mtove G, Nadjm B, Kinabo GD, Philemon R, et al. Invasive salmonella infections in areas of high and low malaria transmission intensity in Tanzania. American Journal of Tropical Medicine and Hygiene. 2013;89: 171. doi:10.1093/cid/cit798

29. Schwarz NG, Sarpong N, Hünger F, Marks F, Acquah SE, Agyekum A, et al. Systemic bacteraemia in children presenting with clinical pneumonia and the impact of non-typhoid salmonella (NTS). BMC Infect Dis. 2010;10: 319. doi:10.1186/1471-2334-10-319

30. Oneko M, Kariuki S, Muturi-Kioi V, Otieno K, Otieno VO, Williamson JM, et al. Emergence of Community-Acquired, Multidrug-Resistant Invasive Nontyphoidal *Salmonella* Disease in Rural Western Kenya, 2009–2013. Clinical Infectious Diseases. 2015;61: S310–S316. doi:10.1093/cid/civ674

31. Luvsansharav UO, Wakhungu J, Grass J, Oneko M, Nguyen V, Bigogo G, et al. Exploration of risk factors for ceftriaxone resistance in invasive non-typhoidal Salmonella infections in western Kenya. PLoS One. 2020;15. doi:10.1371/journal.pone.0229581

32. Appiah GD, Mpimbaza A, Lamorde M, Freeman M, Kajumbula H, Salah Z, et al. Salmonella Bloodstream Infections in Hospitalized Children with Acute Febrile Illness—Uganda, 2016–2019. Am J Trop Med Hyg. 2021 [cited 21 Jun 2021]. doi:10.4269/ajtmh.20-1453

33. Tack B, Vanaenrode J, Verbakel JY, Toelen J, Jacobs J. Invasive non-typhoidal Salmonella infections in sub-Saharan Africa: a systematic review on antimicrobial resistance and treatment. BMC Med. 2020;18. doi:10.1186/s12916-020-01652-4
